# RNA-sequence data normalization through in silico prediction of reference genes: the bacterial response to DNA damage as case study

**DOI:** 10.1186/s13040-017-0150-8

**Published:** 2017-09-05

**Authors:** Bork A. Berghoff, Torgny Karlsson, Thomas Källman, E. Gerhart H. Wagner, Manfred G. Grabherr

**Affiliations:** 1 Institut für Mikrobiologie und Molekularbiologie, Justus-Liebig-Universität, Giessen, Germany; 2 Department of Immunology, Genetics and Pathology, Uppsala University, Uppsala, Sweden; 3 Department of Medical Biochemistry and Microbiology, Uppsala University, Uppsala, Sweden; 4 Bioinformatics Infrastructure for Life Sciences (BILS), Science for Life Laboratories, Uppsala University, Uppsala, Sweden; 5 Department of Cell and Molecular Biology, Uppsala University, Uppsala, Sweden

**Keywords:** RNA-seq, Transcriptomics, Normalization, Gene expression, DNA damage, Stress response

## Abstract

**Background:**

Measuring how gene expression changes in the course of an experiment assesses how an organism responds on a molecular level. Sequencing of RNA molecules, and their subsequent quantification, aims to assess global gene expression changes on the RNA level (transcriptome). While advances in high-throughput RNA-sequencing (RNA-seq) technologies allow for inexpensive data generation, accurate post-processing and normalization across samples is required to eliminate any systematic noise introduced by the biochemical and/or technical processes. Existing methods thus either normalize on selected known reference genes that are invariant in expression across the experiment, assume that the majority of genes are invariant, or that the effects of up- and down-regulated genes cancel each other out during the normalization.

**Results:**

Here, we present a novel method, *moose* ^*2*^, which predicts invariant genes in silico through a dynamic programming (DP) scheme and applies a quadratic normalization based on this subset. The method allows for specifying a set of known or experimentally validated invariant genes, which guides the DP. We experimentally verified the predictions of this method in the bacterium *Escherichia coli*, and show how *moose* ^*2*^ is able to (i) estimate the expression value distances between RNA-seq samples, (ii) reduce the variation of expression values across all samples, and (iii) to subsequently reveal new functional groups of genes during the late stages of DNA damage. We further applied the method to three eukaryotic data sets, on which its performance compares favourably to other methods. The software is implemented in C++ and is publicly available from http://grabherr.github.io/moose2/.

**Conclusions:**

The proposed RNA-seq normalization method, *moose* ^*2*^, is a valuable alternative to existing methods, with two major advantages: (i) in silico prediction of invariant genes provides a list of potential reference genes for downstream analyses, and (ii) non-linear artefacts in RNA-seq data are handled adequately to minimize variations between replicates.

**Electronic supplementary material:**

The online version of this article (10.1186/s13040-017-0150-8) contains supplementary material, which is available to authorized users.

## Background

**Fig. 1:**
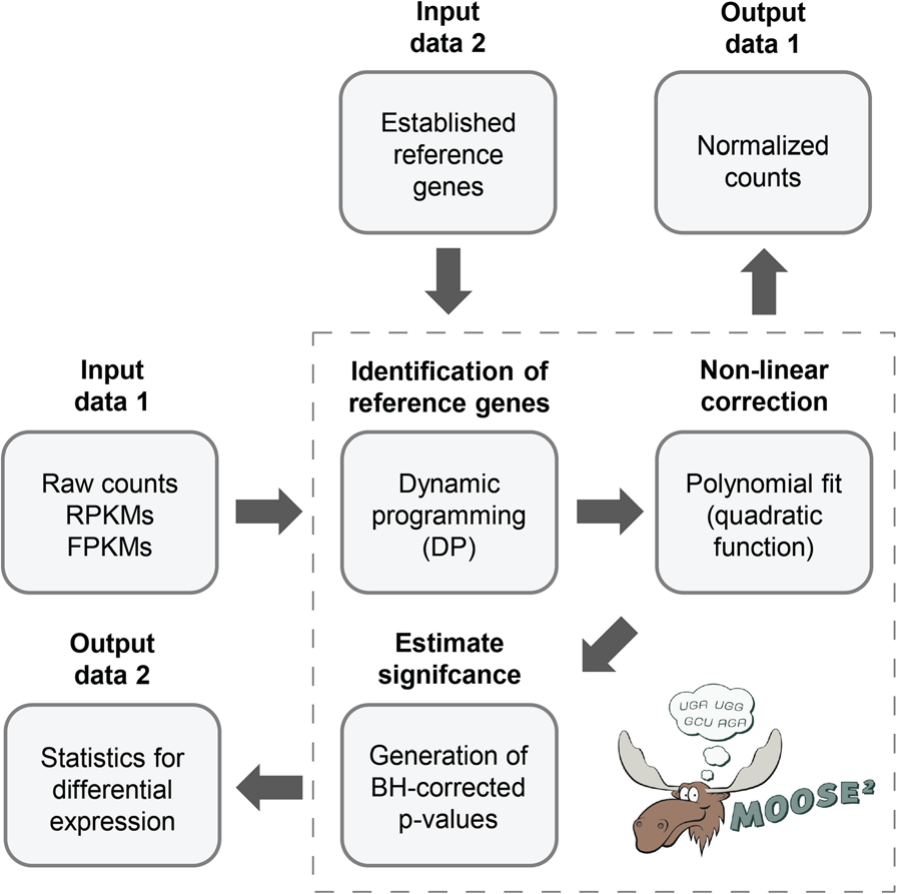
Workflow of *moose* ^*2*^. Input data is presented either as FPKM- or RPKM-normalized values or raw read (or pair) counts, which is specified with the data. If available, a set of known reference genes is provided, which serve as waypoints for the dynamic programming (DP) step. The data is then normalized through a polynomial fit and stored. *Limma* is applied to estimate Benjamini-Hochberg (BH)-corrected *p*-values and the resulting pairwise cross-condition statistics can then be used for expression analyses

RNA-sequencing (RNA-seq) has revolutionized transcriptomics by means of sensitivity, accuracy, and resolution. Additionally, RNA-seq does not rely on prior knowledge of whether any particular RNA is present, and therefore represents a powerful tool for the identification of unknown RNAs. In a typical RNA-seq experiment, total RNA or a particular RNA fraction is isolated from samples that either represent different biological conditions, or replicates from the same condition. After validation of RNA quality, the RNA is subjected to cDNA synthesis via primers that specifically match adapter sequences, or through random priming. If random priming is used, adapter sequences are introduced during subsequent steps. The cDNA is finally amplified by PCR to yield ready-to-use libraries that can be sequenced using different technologies. RNA-seq indirectly measures the abundance of transcripts by the number of reads or fragments generated from a particular transcript. Since the total amount of RNA present in a cell or sample is unknown, data for each sample are either normalized individually by the total read counts per sample and transcript length into RPKM or FPKM values [1], or over all samples by methods such as Upper Quartile (UQ) normalization [2], DESeq2 [3], or Trimmed-Mean of M-values (TMM) normalization [4] (for a review, see ref. [5]). Assumptions underlying the latter methods are that: (i) the mean expression of genes, on which the normalization is computed, does not change across experiments; and (ii) that a single global scaling factor is valid over the entire dynamic range of expression. Importantly, normalization methods may perform poorly if the assumptions do not match the biological experiment [6]. As an alternative to global scaling factors, the use of reference genes, i.e. genes that are invariable in expression regardless of condition or sample, has been suggested [7, 8]. Here, we present a novel method, *moose* ^*2*^ (Fig. 1), which uses known reference genes if available, and additionally predicts reference genes in silico by a dynamic programming (DP) scheme. Application of a polynomial model then allows for normalizing of the entire data set in a non-linear fashion depending on transcript abundance. Hence, our approach specifically aims at satisfying the assumptions that: (i) there is a small, identifiable subset of genes that is not differentially expressed in the given experiment; and (ii) that a quadratic function approximates any non-linear characteristics of expression measurements across the dynamic range.

To validate this method, we examined the bacterial response (in *E. coli*) to the chemotherapeutic drug mitomycin C (MMC), which we investigated at early and late time-points by RNA-seq. MMC is a potent DNA crosslinker that will ultimately generate double-stranded breaks (DSB) in DNA and thereby activate the so-called SOS response. The SOS response is initiated whenever DNA damage occurs. This generates single-stranded DNA (ssDNA) which is bound by the RecA protein. RecA-nucleofilaments subsequently trigger autocleavage of the LexA repressor that controls a regulon of >50 genes in *E. coli*, many of which have functions in DNA repair [9–12]. The response to DNA damage has been intensively studied by microarray analysis of *E. coli* cells that have been treated with UV light, MMC, or quinolone antibiotics [13–17]. However, none of these studies followed the response to high levels of DNA damage over an extended period of time, nor did they capture possible, more subtle changes in gene expression. Here, relative changes in transcript levels were calculated from an RNA-seq study of *E. coli* cells treated with a high dose of MMC for up to 90 min. *Moose* ^*2*^performed better than the other tested normalization methods in terms of (i) the Euclidean distances, which are lower in within-replicate comparisons than in cross-condition comparisons, as is to be expected; and (ii) minimizing the variation of expression values across samples. Thus, the *moose* ^*2*^ results could be used to predict the expression profiles of functional groups, such as the LexA regulon, which gave exciting new insights into the bacterial response after prolonged DNA damage. In addition to this bacterial system, we also applied the approach to three eukaryotic data sets and compared the results to other methods.

## Methods

### Cultivation of bacteria and sampling

*Escherichia coli* MG1655 cells, obtained from CGSC (Coli Genetic Stock Center) at Yale University (http://cgsc.biology.yale.edu) were grown aerobically in Luria broth (LB) at 37 °C. Triplicate overnight cultures were diluted 1:100 into fresh LB and grown for 2 h to reach an OD_600_ of ~0.35. Mitomycin C (MMC) was added at a final concentration of 2.5 μg/ml to induce DNA damage. Samples were withdrawn at 0, 30, and 90 min, and immediately mixed with 0.25 vol of RNA stop solution (95% ethanol, 5% phenol) on ice. Cells were pelleted by centrifugation and frozen in liquid nitrogen. Pellets were thawed on ice and immediately processed for RNA extraction.

### RNA extraction and quality control

Total RNA was prepared by the hot acid-phenol method [18]. Cells were resuspended in lysis buffer (100 mM Tris pH 7.5, 40 mM EDTA, 200 mM NaCl, 0.5% SDS) and incubated at 65 °C for 5 min. After adding acidic phenol (pH 4.0) to the cell lysate, extraction mixtures were incubated at 65 °C for 3 min, frozen in liquid nitrogen, and centrifuged for phase separation. RNA was precipitated from supernatants using isopropanol and pelleted by centrifugation. RNA was washed in 75% ethanol and resuspended in RNase-free water. Samples were treated with DNase I (Thermo Scientific) and extracted with phenol/chloroform, followed by precipitation with isopropanol as before. PCR using primers BB1 (GCT TTA CAG GGG AGA CAA) and BB2 (AAC CCG CAC GCT AAA TAT) was applied to test for DNA contamination. Absorbance ratios of A260/A280 and A260/A230 were determined using a NanoDrop ND-1000 spectrophotometer to assure purity of RNA. RNA integrity was assessed on 1% agarose gels containing 25 mM guanidinium thiocyanate and by analysis with an Agilent 2100 Bioanalyzer (Agilent Technologies) using the Agilent RNA 6000 Pico Kit for total prokaryotic RNA.

### Library preparation, RNA-sequencing and read mapping

#### Preparation of cDNA libraries

Sequencing libraries were prepared with the Encore Complete Prokaryotic RNA-Seq DR Multiplex System (NuGEN) using 200 ng total RNA as input. After cDNA synthesis, cDNA was fragmented by ultra-sonication using the Covaris S-Series System according to the recommendations of the Encore protocol. Adapters containing unique barcode sequences and target sites for Illumina sequencing primers were ligated to cDNA fragments. After strand selection and adapter cleavage, cDNA was amplified to yield strand-specific ready-to-use sequencing libraries. Length distribution of amplified cDNA fragments was validated by Agilent 2100 Bioanalyzer (Agilent Technologies) using the Agilent High Sensitivity DNA Kit. Ultra-sonication and length distribution analysis were performed by the SNP&SEQ Technology Platform in Uppsala, Sweden (www.sequencing.se).

#### Quality control of sequencing libraries

The quality of the libraries was evaluated using the Advanced Analytical Technologies Fragment Analyzer and a DNA-kit (DNF910). The adapter-ligated fragments were quantified by qPCR using the Library quantification kit for Illumina (KAPA Biosystems) on a StepOnePlus instrument (Applied Biosystems/Life Technologies) prior to cluster generation and sequencing.

#### Cluster generation and sequencing

An 11 pM solution of sequencing library was subjected to cluster generation and paired-end sequencing with 100 bp read length on the HiSeq 2500 system (Illumina Inc.) using v3 chemistry according to the manufacturer’s protocols. Base calling was done by RTA 1.17.21.3 and the resulting.bcl files were demultiplexed and converted to fastq format with tools provided by CASAVA 1.8.4 (Illumina Inc.), allowing for one mismatch in the index sequence. Additional statistics on sequence quality were compiled with an in-house script from the fastq-files, RTA and CASAVA output files. Sequencing was performed by the SNP&SEQ Technology Platform in Uppsala, Sweden (www.sequencing.se).

#### Read mapping

Reads were mapped against the *E. coli* K-12 MG1655 genome sequence using Papaya (http://sourceforge.net/projects/satsuma/) from the Satsuma [19] package, and counted against the NCBI GenBank annotations (NC_000913), requiring a minimum alignment length of 60 nt and identity >0.98.

### qRT-PCR

RNA concentrations were determined with a Qubit 2.0 Fluorometer (Invitrogen) using the Qubit RNA HS Assay Kit (Molecular Probes). Primers for qRT-PCR were optimized using primer design software (Additional file 1). The Brilliant III Ultra-Fast SYBR Green QRT-PCR Master Mix (Agilent Technologies) was applied to perform an ultra-fast one-step protocol on a StepOnePlus Real-Time PCR System (Applied Biosystems/Life Technologies) using the following settings for amplification: 50 °C - 10 min, 95 °C - 3 min, 45× (95 °C - 5 s, 60 °C - 10 s). Initial RNA concentrations were set to 1 ng/μl, except for *ssrA* and *rrsA* (set to 10 pg/μl). Melting curves were recorded to monitor amplification specificity. All samples were measured as technical triplicates. Cycle threshold (Ct) values were automatically determined in the linear amplification phase as implemented in the StepOne Software v2.3. Relative fold changes of gene expression were calculated according to the 2^-ΔΔCt^ method [20]. The average Ct values of the six reference genes *cysG*, *idnT*, *hcaT*, *ihfB*, *ssrA*, and *rrsA* were used for normalization.

### Normalization methods

#### Identifying putative invariant genes

In order to increase the number of invariant reference genes, we developed a numerical method to identify such genes in silico. The identification can be regarded as an optimization problem, or more specifically a particular type of linear programming called minimum cost network flow problem [21]. Particularly, the idea is to find the cheapest path in a directed, acyclic *n*_s_-dimensional graph (*n*_s_ denotes the total number of samples), from the most lowly, non-zero expressed gene (source) to the most highly expressed gene (target), given a number of constraints. Any edge (*i,j)* that connects two nodes (genes) in the graph points in the direction from the lower ranked gene to the higher ranked gene, where the ranking, *i = 1,...,n*, is determined by sorting the values of the gene expression averaged over all samples. The genes with identically zero expression are omitted from the analysis. Note that in the graph, each gene is connected to every other gene. The linear program may be written in the form (to indicate the direction in the graph, the first index denotes the lower ranked gene which it is always smaller than the second index, denoting the higher ranked gene)

$$ \mathrm{minimize}\ z=\sum_{i=1}^{n-1}\sum_{j=i+1}^n{c}_{ij}{x}_{ij},\backslash $$

$$ \mathrm{subject}\  \mathrm{to}\ \sum_{j=2}^n{x}_{ij}=1,i=1, $$

1 $$ \sum_{j=i+1}^n{x}_{ij}-\sum_{j=1}^{i-1}{x}_{ji}={b}_i,i=2,\dots, n-1,\kern5em $$

$$ \kern0.5em \sum_{j=1}^{n-1}{x}_{ji}=1,i=n, $$

where *x*_*ij*_ ∈ {0, 1} is the indicator variable that signals whether the edge between the two genes *i* and *j* belongs to the cheapest path (*x*_*ij*_ = 1) or not (*x*_*ij*_ = 0), *c*_*ij*_ is the cost associated with the path connecting gene *i* and *j*, *b*_*i*_ denotes the source or sink at node *i*, while *n* denotes the number of nodes. The cost *c*_*ij*_ is given by

2 $$ \kern7.75em {c}_{ij}={d}_{ij}+\left(j-i-1\right)m+{k}_{ij}h,\kern0.5em j>i,\kern4em $$

where *d*_*ij*_ is the normalised Euclidean distance between gene *i* and *j*, *m* = 4.0 is a flat score introduced as a penalty for taking a path between two genes which are not immediately adjacent in ranking, and *k*_*ij*_ *h* (*h* = 5.0 is constant) denotes the penalty given to those edges for which a number of *k*_*ij*_ samples of the higher ranked gene *j* have a lower expression (RPKM) than the corresponding samples of the lower ranked gene *i*. Finally, the sources and sinks of the interior nodes *i* = 2,...,*n* − 1 are given by.

$$ {b}_i=\left\{\begin{array}{c}-r,\kern0.5em i=\mathrm{ranking}\  \mathrm{of}\  \mathrm{known}\  \mathrm{reference}\  \mathrm{gene}\\ {}0,\kern2.25em \mathrm{otherwise}.\kern12.25em \end{array}\right. $$

In order to speed up the computation, the linear program in Eq. (1) is solved by applying a dynamic programming algorithm. Also, in this way, the costs *c*_*ij*_, given by the expression in Eq. (2), do not have to be pre-computed. The specific values of the reward sinks *b*_*i*_ =  − *r* =  − 800 (*r* ≫ 1 in order to ensure that known reference genes are included in the path), which is roughly the number of genes divided by the number of reference genes. The penalties *m* = 4.0 and *h* = 5.0, which control the number of identified in silico reference genes, are chosen to produce about 30 reference genes that are roughly evenly spaced in log(expression) space (for parameter choice, see Additional file 2 and the *moose* ^*2*^ manual on the web site at http://grabherr.github.io/moose2/. In case no reference genes are provided, the algorithm selects the statistically best estimate from the experimental data.

#### Non-linear (polynomial) correction

For each individual sample, we fit a linear model of second order (i.e., a parabola), such that

3 $$ {y}_i={\beta}_0+{\beta}_1{x}_i+{\beta}_2{x}_i^2+{\varepsilon}_i,\kern2em i=1,\dots, n,\kern11em $$

based on the *n* in silico invariant genes (including any pre-defined house-keeping genes). In (3), *x*_*i*_ denotes the logarithm of the RPKM-value of invariant gene *i* for the specific sample in question, while *y*_*i*_ denotes the mean of the log(RPKM) of gene *i,* taken over all samples, and *ε*_*i*_ denotes the error term.In matrix notation, (3) may be written as ***Y*** = ***Xβ*** + ***ε*** and the ordinary least-squares (OLS) estimates, $\widehat{\boldsymbol{\beta}}={\left({\widehat{\beta}}_0,{\widehat{\beta}}_1,{\widehat{\beta}}_2\right)}^T$, of the model parameters are then simply given by $\widehat{\boldsymbol{\beta}}={\left({\boldsymbol{X}}^{\boldsymbol{T}}\boldsymbol{X}\right)}^{-1}{\boldsymbol{X}}^{\boldsymbol{T}}\boldsymbol{Y}$, where the sum of squared residuals

$$ Q={\sum}_{i=1}^n{\left({y}_i-\left({\beta}_0+{\beta}_1{x}_i+{\beta}_2{x}_i^2\right)\right)}^2 $$

have been minimized s.t. ∂*Q*/∂*β*_0_ = ∂*Q*/∂*β*_1_ = ∂*Q*/∂*β*_2_ = 0. This fit is then used to assign a corrected log-RPKM value,

$$ {\widehat{y}}_k={\widehat{\beta}}_0+{\widehat{\beta}}_1{x}_k+{\widehat{\beta}}_2{x}_k^2, $$

to each gene *k*, based on the gene’s observed log-RPKM value (*x*_*k*_) for the sample in question. Note that the estimates $\widehat{\boldsymbol{\beta}}={\left({\widehat{\beta}}_0,{\widehat{\beta}}_1,{\widehat{\beta}}_2\right)}^T$ will be different for each sample.

#### Implementation

The algorithms are implemented in C++ and are publicly available from https://github.com/grabherr/moose2 under the General Public License (GPL).

#### Linear (global) normalization

For linear normalization we used four different algorithms: RPKM [1], UQ [2], DESeq2 [3], and TMM [4]. DEseq2 and TMM normalization were performed using the R statistical language (http://www.r-project.org/) and Bioconductor (http://www.bioconductor.org/) packages ‘DESeq2’ [3] and ‘edgeR’ [22]. In the ‘DESeq2’ package, functions *estimateSizeFactors* and *sizeFactors* were called to receive sample-specific normalization factors. In the ‘edgeR’ package, function *calcNormFactors* was called to output scaling factors together with the original library size. The product of the original library size and the scaling factor, the so-called effective library size, was used as a sample-specific normalization factor. All fold-changes were calculated as direct log_2_ ratios from the normalized read counts.

#### Estimating *p*-values

For identification of differentially expressed genes the *moose* ^*2*^-corrected counts were first transformed using the voom function then subjected to the actual gene expression analysis using *Limma* [23]. In short, voom estimates mean-variance relationships of log-counts for the samples and *Limma* identifies differentially expressed genes in a linear modelling framework using an empirical Bayesian method to moderate standard errors and estimate fold changes between conditions. From these values, moderated t-statistic and the corresponding *p*-values are estimated. Finally, Benjamini-Hochberg correction of *p*-values was used to control the false discovery rate.

### Cluster analyses

Hierarchical cluster analysis of RNA-Seq samples was performed using the R statistical language (http://www.r-project.org/). Function *dist* (method = euclidean) was called to generate distance matrices based on log_2_-transformed read counts (with a pseudocount of 1). Alternatively, the ‘DESeq2’ package provides log-transformation schemes that are based on per-gene dispersion estimates. Expression data, normalized by DESeq’s *sizeFactors*, were applied to the regularized log transformation (function *rlog*) and variance stabilizing transformation (VST; function *varianceStabilizingTransformation*), using dispersion estimates calculated with function *estimateDispersions*. Distance matrices were subsequently generated. All distance matrices were used as input for function *hclust* (method = ward.D2) to generate cluster trees.

Expression cluster analysis of time-series data based on fuzzy *c*-means was performed using the R statistical language (http://www.r-project.org/) and Bioconductor (http://www.bioconductor.org/) package ‘Mfuzz’ [24]. For soft clustering of the Top-1000 list (see main text), the fuzzification parameter was set to *m* = 2 and the number of clusters to *c* = 6. Results are displayed in Additional file 3.

Functional annotation clustering of genes was performed using the DAVID bioinformatics database [25] (http://david.abcc.ncifcrf.gov/home.jsp). Medium classification stringency with default settings was used to generate clusters of gene ontology (GO) terms based on biological process (BP), cellular component (CC), and molecular function (MF). Pathway clustering was performed in a similar way using the KEGG (http://www.genome.jp/kegg/) pathway option. Results are displayed in Additional file 4.

### RNA-seq data

The original RNA-seq datasets for *E. coli* are distributed with *moose* ^*2*^ (https://github.com/grabherr/moose2). *Moose* ^*2*^-normalized data including significance analysis can be found as Additional file 5.

## Results

### Distortion of RNA-seq read counts depends on transcript abundance

**Fig. 2:**
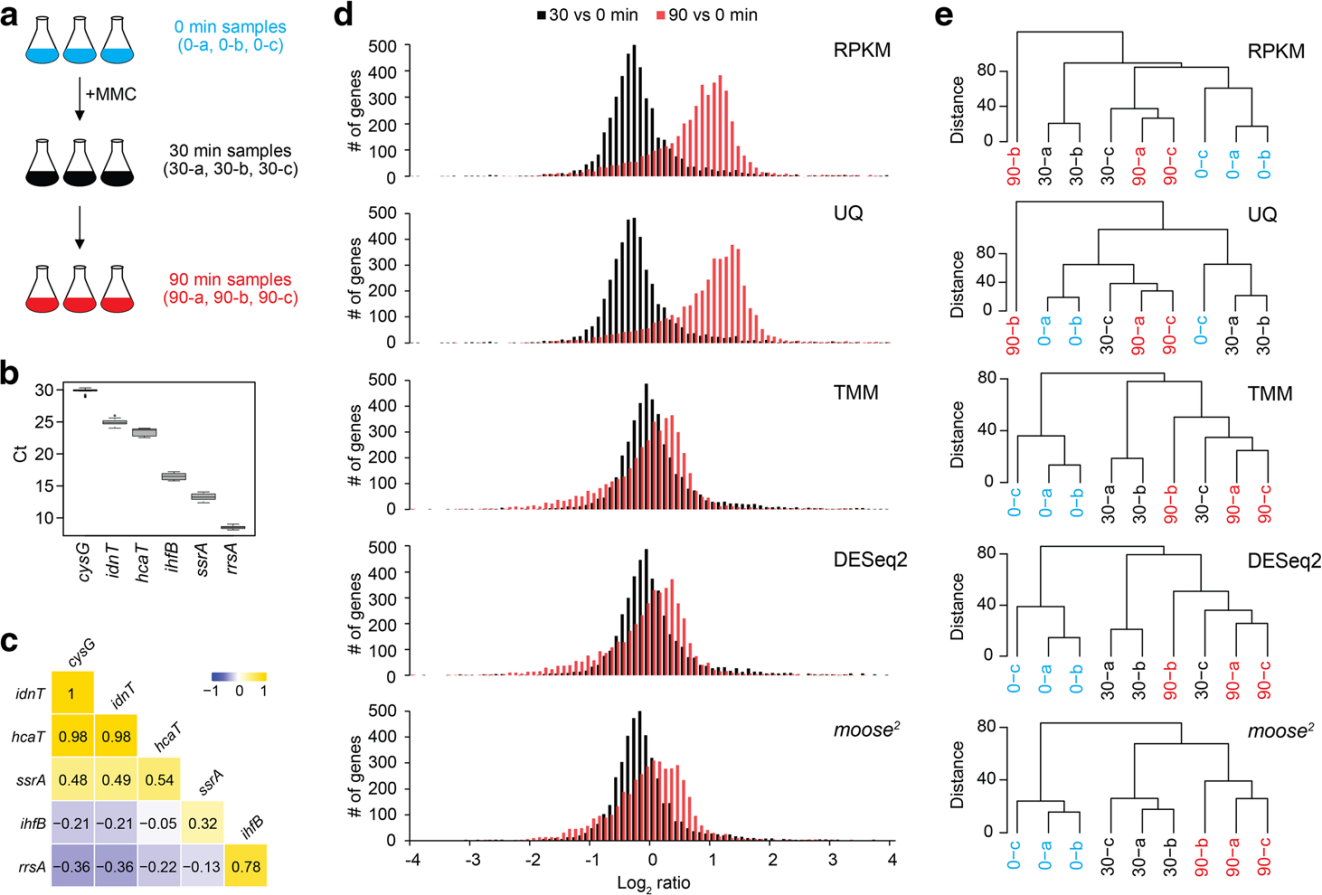
Experimental design and evaluation of *moose* ^*2*^. **a** Triplicate *E. coli* MG1655 cultures were treated with MMC, and total RNA samples were sequenced on an Illumina platform. **b** Variation plot for expression of six established reference genes as measured by qRT-PCR. Ct, cycle threshold. **c** Correlation between six established reference genes over all non-normalized RNA-seq samples, sorted by transcript abundance. The lowly expressed *cysG*, *idnT*, and *hcaT* are positively correlated (shown in yellow), but negatively correlated with the more abundant *ihfB* and *rrsA*. The colour scale bar reflects Pearson’s rho. **d** Log_2_-ratio distribution in cross-condition comparisons, shown for RPKM, Upper Quartile (UQ), Trimmed-Mean of M-values (TMM), DESeq2, and *moose* ^*2*^. **e** Hierarchical clustering based on the Euclidean distance between RNA-seq samples. Matched conditions indicated as blue (0 min), black (30 min), and red (90 min)

We performed an experiment in which we exposed *E. coli* cells (strain MG1655) to high doses of MMC and measured gene expression at 0 (control), 30, and 90 min in three biological replicates each by Illumina sequencing (Fig. 2a). Earlier microarray-based studies on the SOS response, using UV light or MMC, showed a global change in gene expression, but might have missed effects after prolonged time of severe DNA damage [13, 14]. To establish a set of reference genes, we monitored mRNA levels of the widely used housekeeping genes *ihfB*, *ssrA*, and *rrsA*, as well as expression of recently suggested reference genes *cysG*, *idnT*, and *hcaT* [26] by qRT-PCR. Figure 2b indicates that the reference genes were expressed at stable levels and not subject to systematic bias (Additional file 6). However, calculating the pairwise correlations of the six reference genes over all non-normalized RNA-seq samples suggested that even though the moderately expressed *cysG*, *hcaT*, and *idnT* levels were positively correlated (Pearson’s rho ≥0.98), the estimated correlation with the highly expressed *rrsA* and *ihfB* was negative (Pearson’s rho ≤ −0.05, Fig. 2c). Thus, the presence of a systematic bias likely distorts the expression measurements by RNA-seq in dependence of transcript abundance.

### In silico reference genes allow for non-linear transformation of expression values

**Fig. 3:**
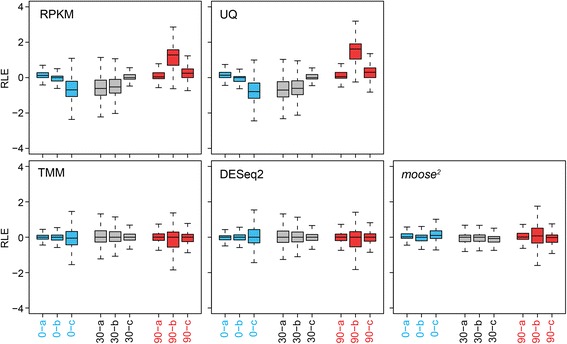
*Moose* ^*2*^ reduces the variation across samples. Gene expression values (read counts) from individual samples were compared to the median across all samples, and changes calculated as log_2_ ratios. Relative log expression (RLE) boxplots represent the distribution of log_2_ ratios, and are shown for RPKM, Upper Quartile (UQ), Trimmed-Mean of M-values (TMM), DESeq2, and *moose* ^*2*^

We first applied the RPKM, UQ, TMM and DESeq2 normalization methods, and plotted the log_2_-ratio distributions comparing the combined replicates across time-points (Fig. 2d). We next computed a distance matrix based on the respective Euclidean distances of all genes (as log_2_-transformed expression values) across samples, and performed hierarchical clustering (Fig. 2e). Notably, none of the normalization schemes correctly grouped all samples by experiment, indicating that the distances are not consistently lower in within-replicate comparisons. Moreover, reducing the variance between samples by using per-gene dispersion estimates for the log-transformation (e.g., *rlog* and VST in the ‘DESeq2’ package) did not reproduce the correct grouping of biological replicates (Additional file 7). Processing the data with *moose* ^*2*^, guided by the six established reference genes (Fig. 2b), predicted 27 additional in silico reference genes (Table 1. For a more detailed analysis on how selecting in silico genes depends on the choice of conditions, see Additional file 8). While TMM, DESeq2 and *moose* ^*2*^ estimate the peaks of the cross-experiment comparisons around the same position (Fig. 2d), *moose* ^*2*^ reduces the tails on both sides in the 90-to-0-min distribution. Moreover, Euclidean distances based on *moose* ^*2*^-normalized expression values correctly resolve the grouping of biological replicates (Fig. 2e). Notably, the coefficients for the quadratic correction term, ranging from −0.044 to 0.029, were weakly inversely correlated (Pearson’s rho = −0.72, *p* < 0.018) with the total number of raw reads of each sample, possibly indicating that nonlinearity could have been introduced during library construction, e.g. during random priming, or in the sequencing process. Finally, accurate data normalization is expected to reduce the variation of expression values across all samples. Relative log expression (RLE) boxplots represent the distribution of log_2_ ratios for all genes between one particular sample and the median across all samples. The RLE boxplots should be ideally centered on zero and exhibit a similar dispersion. In contrast to RPKM and UQ normalization, TMM and DESeq2 shifted the mean values close to zero, but without major effect on the variation, while *moose* ^*2*^ clearly reduced the variation (Fig. 3).

**Table 1:** Expression-invariant genes in *E. coli* during DNA damage as identified by the DP scheme and used for *moose* ^*2*^

Gene	Product	Process/Function
*cysG* ^a^	uroporphyrin III C-methyltransferase	Other
*dnaG*	DNA primase	DNA replication
*dtpB*	dipeptide/tripeptide:H^+^ symporter DtpB	Transport
*ftsX*	Cell division protein ftsX	Cell division
*ftsY*	Cell division protein ftsY	Cell division
*glyY*	tRNA_glyY_	Translation
*gyrB*	DNA gyrase, subunit B	DNA replication
*hcaT* ^a^	putative transport protein, major facilitator superfamily (MFS)	Transport
*idnT* ^a^	L-idonate / 5-ketogluconate / gluconate transporter IdnT	Transport
*ihfB* ^a^	integration host factor (IHF), beta subunit	Transcription
*lhr*	member of ATP-dependent helicase superfamily II	DNA replication
*mutM*	formamidopyrimidine DNA glycosylase	DNA repair
*mutY*	A/G-specific adenine glycosylase	DNA repair
*ndk*	nucleoside diphosphate kinase	Other
*nfuA*	iron-sulfur cluster scaffold protein	Other
*pnp*	polynucleotide phosphorylase monomer	RNA processing
*rbbA*	ribosome-associated ATPase	Translation
*rhsB*	RhsB protein in *rhs* element	Other
*rpsU*	30S ribosomal subunit protein S21	Translation
*rrsA* ^a^	16S ribosomal RNA (*rrsA*)	Translation
*rrsE*	16S ribosomal RNA (*rrsE*)	Translation
*rrsG*	16S ribosomal RNA (*rrsG*)	Translation
*secB*	SecB chaperone	Protein localization
*spoT*	Guanosine-3′,5′-bis(diphosphate) 3′-pyrophosphohydrolase	Other
*ssrA* ^a^	tmRNA	Trans-translation
*tfaR*	Rac prophage; predicted tail fiber assembly protein	Prophage
*thrW*	tRNA_thrW_	Translation
*valS*	Valyl-tRNA synthetase	Translation
*yedJ*	predicted phosphohydrolase	Other
*ynaE*	Rac prophage; cold shock protein, function unknown	Prophage
*yphG*	conserved protein	Unknown
*zntA*	zinc, cadmium and lead efflux system	Transport
*zupT*	heavy metal divalent cation transporter ZupT	Transport

^a^Established reference genes in *E. coli* (see main text and Fig. 2b) were used as an input for *moose* ^*2*^

We next investigated the individual contributions stemming from (a) predicted in silico reference genes; (b) the quadratic correction term; and (c) guiding the in silico prediction by established reference genes. We thus eliminated each feature individually, and found that the sample grouping was only correct when including both in silico prediction and non-linear correction based on the quadratic term (Additional file 9). To verify, we normalized the data with DESeq2 based on (a) the six established reference genes, and (b) the 33 predicted invariant genes, confirming that a non-linear correction term is required for correct sample grouping (Additional file 10).

To assess the role of accurate reference genes, we ran *moose* ^*2*^ with (a) six genes that were randomly selected over the expression range; and (b) the six most differentially expressed genes (Additional file 9). Interestingly, the resulting sample groupings are correct even in the second case, due to *moose* ^*2*^ rejecting five out of the six genes as too costly to use in the dynamic programming step, reverting to a different set of predictions. In either experiment, however, the RLE boxplots are not as closely centered on zero and/or the variance is larger than when incorporating the six established reference genes used in the full analysis (Additional file 11).

### In-silico predicted expression-invariant transcripts contain housekeeping genes

Genes with stable expression patterns across a variety of conditions often serve housekeeping functions, such as transcription, translation, or replication. The DP scheme in the *moose* ^*2*^ pipeline predicted 27 expression-invariant genes, many of which have indeed housekeeping functions (Table 1). The most dominant group comprises genes with a function in translation, including ribosomal RNAs, one ribosomal protein, transfer RNAs, and one aminoacyl tRNA synthetase. Furthermore, the predicted invariant genes were enriched for functions in DNA replication and repair. These findings support the accuracy of the DP scheme for the identification of reference genes.

We tested the 33 reference genes (six established and 27 predicted) for their correlation to each other across all RNA-seq samples. Since normalization is expected to reduce systematic errors in read counts, such as library size effects, one would expect more unstructured correlation patterns for expression-invariant genes after normalization. While global normalization (e.g. by DESeq2; Additional file 12) produced a structured correlation pattern with many estimated coefficients (Pearson’s Rho) clearly divergent from zero, *moose* ^*2*^ produced a less structured pattern, which would be expected, and more coefficients close to zero (Additional file 13). This analysis suggested that systematic biases are efficiently reduced by *moose* ^*2*^.

### Accurate prediction of expression changes reveals new features of the bacterial response to DNA damage

For further validation, we selected 19 *E. coli* genes, eight being members of the SOS response, for qRT-PCR, widely accepted as the gold-standard for assessing gene expression changes [2, 27]. While the Pearson correlation coefficients between qRT-PCR and *moose* ^*2*^ were comparable to the TMM and DESeq2 methods (Additional file 14), linear regression showed that the constant term, describing the global shift of the data, was closest to zero for *moose* ^*2*^ (−0.072 and −0.021 respectively), compared to TMM (0.264 and −0.381) and DESeq2 (0.204 and −0.413). Even though expression ratios predicted by *moose* ^*2*^ might be slightly underestimated in some cases (Additional file 14), the overall qRT-PCR results were reliably reproduced by *moose* ^*2*^.

**Fig. 4:**
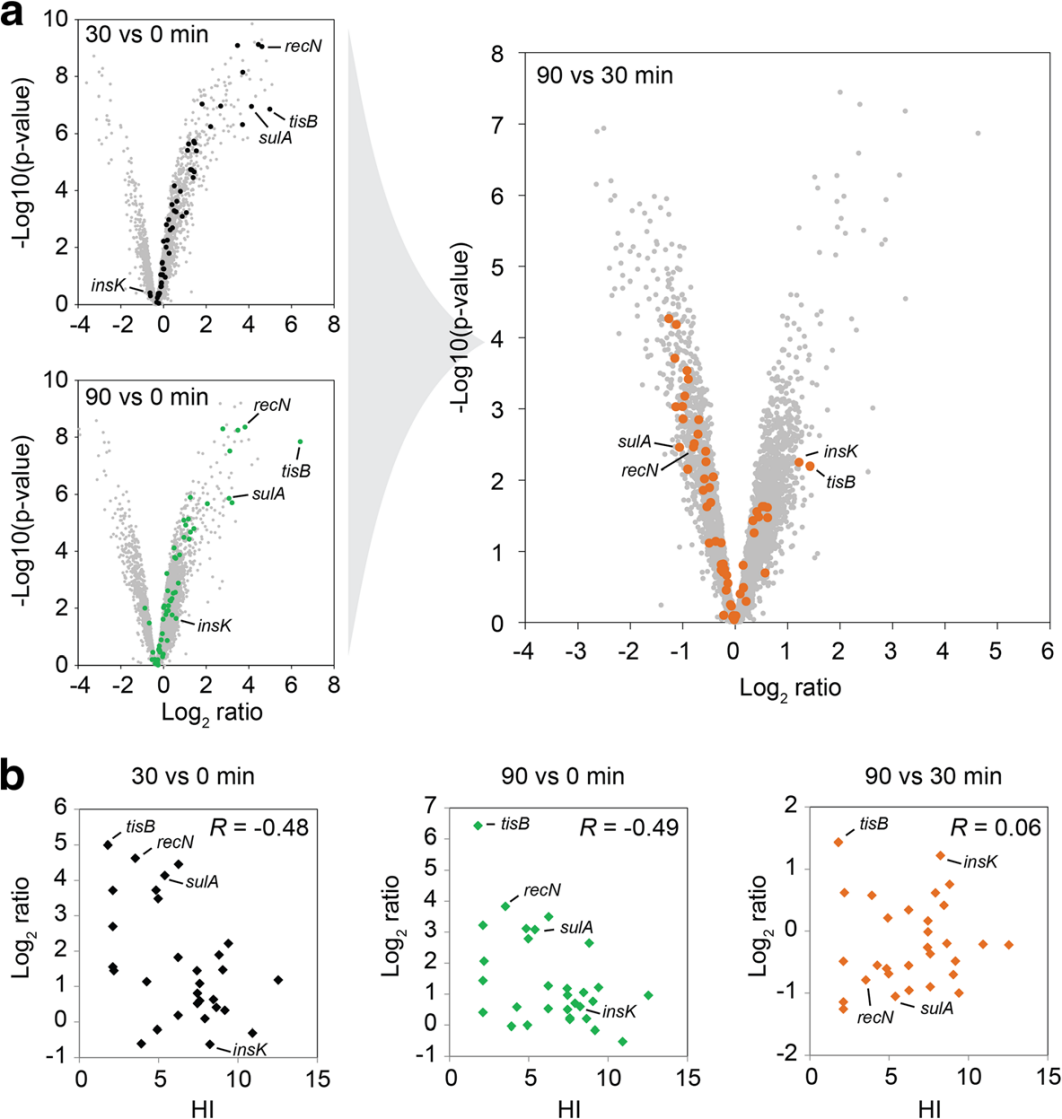
Expression profile of LexA-dependent genes. **a** Volcano plots depicting log_2_ ratios and *p*-values (negative log10) for different comparisons. The 58 LexA-dependent genes are highlighted in black (30-to-0-min comparison), green (90-to-0-min comparison), and orange (90-to-30-min comparison). **b** The log_2_ ratios of 30 LexA-dependent genes were correlated with their respective heterology index (HI) as calculated by Courcelle and colleagues [14]. *R*, Pearson’s rho

We subsequently used *moose* ^*2*^ to globally predict changes in gene expression. From microarray analyses, it is known that ~30 LexA-dependent genes are induced upon UV irradiation [14], and in total more than 1000 genes might be affected by DNA damage [13]. We sorted our expression data according to *p*-values computed by *Limma* to visualize coverage of LexA-dependent genes. According to the RegulonDB database [28], 58 genes might be LexA-dependent. A *p*-value cutoff of *p* < 9.563*10^−4^ generated a list of 1000 genes (Top-1000), representing ~24% of the whole data set, including 31 LexA-dependent genes (Additional file 15). We therefore considered the Top-1000 list as a reliable resource for functionally relevant features and examined the directions in which expression changes between time-points, starting with LexA-dependent genes. Several genes were clearly up-regulated upon MMC treatment as exemplified by *recN*, *sulA*, and *tisB* (Fig. 4a). There were however genes (e.g. *insK*) that responded to MMC only after 90 min, which motivated us to calculate expression changes for the 90-to-30-min comparison. Interestingly, most LexA-dependent genes exhibited reduced transcript levels in this comparison, as observed for *recN* and *sulA*. By contrast, only two LexA-dependent genes were found in the same comparison to be clearly increased at the transcript level (log_2_ ratio > 1). This applied to the toxin gene *tisB* and the putative transposase gene, *insK* (Fig. 4a). Most LexA-dependent genes are preceded by a LexA-box sequence. The heterology index (HI) defines the similarity of a particular LexA-box to the consensus of all LexA-box sequences [10]. Low HI values represent high similarity to the consensus. We compared the log_2_ ratios of 30 genes to their corresponding HI values [14], and found an inverse correlation for the 30-to-0-min and 90-to-0-min comparisons as expected (Pearson’s Rho of −0.48 and −0.49 respectively; Fig. 4b). In contrast, there was no correlation between log_2_ ratios and HI values for the 90-to-30-min comparison (Pearson’s Rho of 0.06), suggesting that changes on transcript level between 30 and 90 min of MMC treatment do not depend on LexA.

**Fig. 5:**
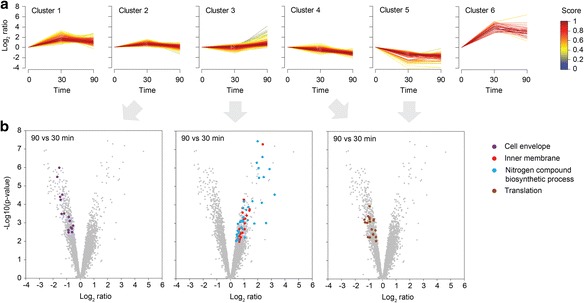
Identification of differentially regulated gene groups after prolonged DNA damage. **a** Soft clustering profiles for the Top-1000 regulated genes (see main text). The colour scale bar reflects the membership score (right). **b** Volcano plots depicting log_2_ ratios and *p*-values (negative log10) for the 90-to-30-min comparison. The different plots highlight functional groups specifically enriched in one of the expression clusters (see legend for details)

The Top-1000 list was applied to soft clustering to generate six expression clusters (Fig. 5a and Additional file 3). LexA-dependent genes were mainly found in expression clusters that exhibited induction at time-point 30 min (clusters 1, 2, and 6). Functional annotation clustering of gene ontology (GO) terms was applied to identify cellular functions that are enriched in distinct expression clusters (Additional file 4), with a focus on the 90-to-30-min comparison (Fig. 5b). Genes with a function in the cell envelope, as e.g. cell wall biosynthesis [*mltB* (murein transglycosylase B) and *oppB* (subunit of murein tripeptide ABC transporter)] or sugar import [*ptsG* (glucose PTS permease) and *manY* (mannose PTS permease)], were decreased in expression after prolonged MMC treatment. The same applied to several genes encoding ribosomal proteins, tRNAs, and aminoacyl tRNA synthetases (Fig. 5b). By contrast, genes with a function intrinsic to the inner membrane or in nitrogen compound biosynthetic processes were only induced at time-point 90 min, as already observed for e.g. *insK*. Among those, several genes encode transporters, and pathway analysis further highlighted genes with a role in purine, amino acid, and sulfur metabolism (Additional file 4).

### *Moose*^*2*^ can be applied to complex eukaryotic samples

**Fig. 6:**
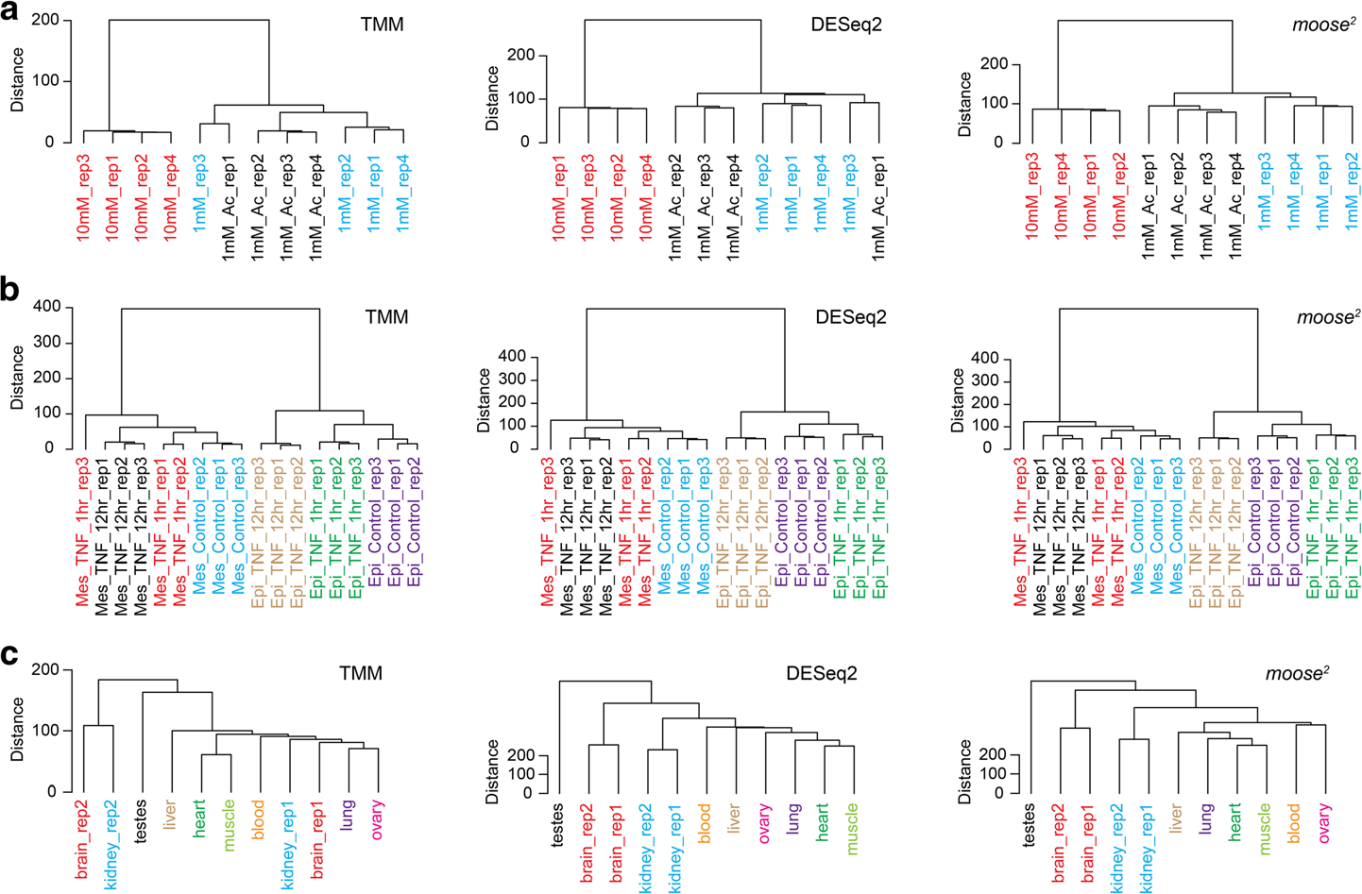
Validation on three eukaryotic data sets. **a** Dendrograms based on the TMM, DESeq2 and *moose* ^*2*^ methods on data from human glioblastoma cells [29] show that *moose* ^*2*^ groups all samples correctly, whereas DESeq2 and TMM place one sample incorrectly. **b** On data from primary airway epithelial cells [30], all three methods result in the same sample grouping, with one outlier sample. **c** On data from nine dog tissues [31] for non-coding, single exon genes, *moose* ^*2*^ and DESeq2 correctly group the replicates and related tissues (heart, muscle)

We next explored whether *moose* ^*2*^ outperforms other methods, such as TMM and DESeq2, on data sets beyond cultured bacteria, by applying it to three eukaryotic data sets. On expression data generated to investigate the glucose- and acetate-regulated transcripts in human glioblastoma cells [29], *moose* ^*2*^ groups all samples correctly, whereas DESeq2 and TMM incorrectly place one sample each (Fig. 6a). On data examining the TGFβ-induced program in human primary airway epithelial cells [30], all three methods result in the same sample grouping, with one sample, Mes_TNF_1hr_rep3, placed outside its clade (Fig. 6b). However, comparing scatter plots between two replicate pairs indicate that sample Mes_TNF_1hr_rep3 is an outlier with a higher variance in expression (data not shown), and that this sample is likely not suitable for a completely accurate analysis. Lastly, on data from nine dog tissues [31] with two sample replicates, *moose* ^*2*^ and DESeq2 correctly group the subset of single-exon, non-coding transcripts, which have been reported to exhibit sample-specific expression patterns, by replicates and related tissues (heart, muscle), with the exception of liver, which appears related to kidney in the expression of protein-coding genes [31] (Fig. 6c).

## Discussion

Adequate normalization of RNA-seq data is an essential step required to reliably predict differentially expressed genes [2]. The correct choice of a normalization method depends on the assumptions that are valid for the particular biological system under investigation. For example, the RPKM method (normalization by library size) assumes that the RNA amount per cell is not changed between conditions, an assumption that is easily violated by differential expression of highly expressed genes [2, 5]. Methods such as DESeq2 and TMM assume that the number of up- and down-regulated genes are balanced between conditions, i.e. expression changes are symmetric. These methods might, therefore, perform poorly when the symmetry of expression changes is skewed towards one direction [6]. The genomes of bacteria comprise relatively few genes (e.g. *E. coli:* ~4500) compared to other organisms, thus any response to outside stimuli might involve a large fraction of genes. This can be exemplified by bacteria exposed to extreme stresses [13, 32] or environments such as macrophages [33, 34], where hundreds of genes are differentially expressed, representing up to one fourth of the whole genome. Even though expression changes are not necessarily asymmetric, many experiments show a clear trend towards either side of regulation. As an alternative to the aforementioned methods, expression data can be normalized by applying control genes, which are either external controls (spike-ins) [7, 35, 36], or invariant genes [2, 37]. The underlying assumption for the latter is that at least a small number of genes exist that are not subject to changes in expression in the given experiment. Here, we applied a new method, *moose* ^*2*^, which first identifies such a small subset of invariant genes in silico, and further applies different normalization factors based on the expression strength of each individual gene. In an experiment exposing *E. coli* to high doses of the DNA damaging agent MMC for an extended period of time, global changes in gene expression can be expected [13, 15], and were validated here (Additional file 16). Importantly, gene expression changes might not be symmetric, since the log_2_ ratio distributions (Fig. 2d) are skewed towards up- and down-regulation for the 30-to-0-min and 90-to-0-min comparisons, respectively. The assumption of symmetric gene expression changes is therefore unjustified, thus necessitating an approach that relies on a different assumption, as the existence of invariant genes. The dynamic programming step in the *moose* ^*2*^ pipeline, guided by six established reference genes, predicted 27 additional genes to be expressed at stable levels across experiments. The majority of these invariant genes are known to perform housekeeping functions. We do note, however, that the term “invariant” only applies to genes that do not change expression in any given experiment, so that the selection of these genes depends on the conditions. While the prediction of in silico genes appears stable in the experiments presented here, we caution that there are cases in which this scheme might perform poorly, notably when analyzing large numbers of conditions, or expression across species. Furthermore, in case invariant genes do not exist, the main assumption of *moose* ^*2*^ is violated and alternative methods are preferable. For example, global changes in gene expression, where most of the genes are up-regulated, have been observed in tumor cells and termed transcriptional amplification [38]. In this special case, invariant genes do not exist and external controls (spike-ins) are needed for adequate normalization of RNA-seq data: in the study of Lovén et al. [35] cyclic loess normalization on the spike-ins was successfully applied. Hence, there are limitations to the usage of *moose* ^*2*^, even though it is expected to perform well for most experimental settings. However, the experimentalist should in every case carefully check the justification of the assumptions before deciding on a normalization method.

Reduction of in-between replicate variation by non-linear correction schemes has already been suggested for microarray experiments [39–41], and our data indicate the general strength of such methods in removing technical bias in expression data as well. Interestingly, for our *E. coli* data set, we found that the quadratic correction term used in the *moose* ^*2*^ pipeline performs best, when based on in silico prediction of invariant genes (Additional file 9). The in silico prediction step is clearly a reasonable basis for a non-linear transformation. The accuracy of subsequently calculated log_2_ ratios was experimentally verified through qRT-PCR for selected genes (Additional file 14). *Moose* ^*2*^, therefore, allows for precisely identifying differentially expressed genes, e.g. using *Limma* [23], or other methods that are continuously being developed and refined in parallel to advances in RNA-seq technologies [42].

The costs for high-throughput sequencing have rapidly decreased over the years, and sequencing of any prokaryotic or eukaryotic organism has become achievable. However, for many organisms, well-annotated reference genomes are unavailable. De novo transcriptome assembly represents an attractive strategy to assess non-sequenced organisms, despite being a bigger informatics challenge than reference-based transcriptome assembly [43, 44]. Furthermore, for downstream analyses such as qRT-PCR, robust reference genes are often needed, but generally not known for non-sequenced organisms. Identification of invariant genes by *moose* ^*2*^ might help to establish reference genes for accurate normalization of qRT-PCR [45]. Different methods have been described for data-driven identification of reference genes [37], and ideally, these methods should be combined with *moose* ^*2*^ to define a reliable set of reference genes. We predict that applying de novo transcriptome assembler together with reference gene identification will benefit the establishment of new model organisms.

As a showcase, we used the *moose* ^*2*^ approach to investigate the response of *E. coli* to prolonged DNA damage caused by MMC. Since there are many treatments that can evoke DNA damage, like ionizing radiation, UV light, DNA gyrase inhibitors, and DNA crosslinkers, the gene expression changes presented here are considered as the MMC-specific response to DNA damage. Also, in a comparative study, aiming to define a global network scheme based on compilations of microarrays, it was found that the SOS response is the only transcriptional response that is consistently triggered upon DNA damage regardless of the toxic agent [16]. As expected and observed here, the degree of induction of several SOS response genes relies on the HI value of the corresponding LexA-box: the lower the HI value, the higher the induction (Fig. 4b). The 90-to-30-min comparison however revealed that most LexA-dependent genes clearly decrease in expression level at the late time-point, which cannot be attributed to their HI values. Since most of the LexA-dependent genes solely depend on LexA and Sigma70 for transcription [28], it is likely that transcript stability and other post-transcriptional mechanisms are pivotal. The strong expression increase of the toxin gene *tisB* (Fig. 4a) is of particular interest, since TisB targets the inner membrane to impair the proton motive force, which then contributes to persister cell formation under DNA-damaging conditions [46–49]. Persisters are transiently drug-tolerant cells that are arrested in their growth due to the action of toxins. The TisB-dependent growth arrest might be accompanied by downstream expression changes, relevant to the persister phenomenon. Gene expression at an early time-point of DNA damage (here 30 min) generally represents the effort to counteract the stressful condition, i.e. inhibiting cell division and repairing DNA damages. The situation changes at late stages (here 90 min), when a fraction of cells has experienced a high level of DNA damage and consequently died, while the surviving subpopulation (i.e. persisters) have only faced moderate DNA damage [50]. So, the SOS response is expected to decline, and this is exactly what we observe (Fig. 4a). Since our RNA-seq data are based on bulk experiments, conclusions have to be drawn cautiously, and some of the gene expression changes at 90 min may reflect the surviving subpopulation. The clear up-regulation of transporters and enzymes involved in purine and amino acid metabolism (Fig. 5b) might factor into long-term survival strategies of the bacteria. Interestingly, the same GO terms have been found to be under-represented during short periods of DNA damage [16], and might therefore be highly specific to late adaptation processes.

## Conclusions

In summary, we present a novel method, *moose* ^*2*^, and show that it corrects for systematic bias in RNA-seq expression data from a bacterial data set by normalizing expression values against a set of genes that were predicted as invariant in silico. Moreover, when applied to more complex eukaryotic data sets, the method performs consistently as well as, or better than, other RNA-seq normalization methods, indicating that its algorithm is also applicable to a wider set of organisms. The software is modular and can easily be integrated with other methods that require a set of invariant genes for normalization. *Moose* ^*2*^ is written in C++ and freely available as source code under the General Public License from http://grabherr.github.io/moose2/.

## Additional files

Additional file 1: Primers used for qRT-PCR. (PDF 18 kb)

Additional file 2: Number of in silico invariant genes depending on the choice of parameters. The number of genes depends on *h* and *m*, with several settings resulting in the same set of 33 genes used in this analysis. (DOCX 8 kb)

Additional file 3: Expression cluster analysis of time-series data. The data sheet contains the results for the expression cluster analysis of time-series data of the Top-1000 list. Bioconductor package ‘Mfuzz’ was applied for soft clustering. (XLSX 47 kb)

Additional file 4: Functional annotation cluster analysis. Expression clusters as determined by soft clustering were applied to functional annotation clustering using the DAVID bioinformatics database. The data sheet contains the results for gene ontology (GO) terms (BP: biological process; CC: cellular component; MF: molecular function) and pathway analyses (KEGG). (XLSX 288 kb)

Additional file 5: *Moose* ^*2*^-normalized RNA-seq data. The data sheet contains the RNA-seq read counts after *moose* ^*2*^ normalization and *p*-values for cross-condition comparisons. (XLSX 723 kb)

Additional file 6: Box plots of reference genes broken down by condition. While there are minor differences in expression levels, there is no systematic trend in either direction. (TIFF 238 kb)

Additional file 7: Per-gene dispersion estimates for the log-transformation. Using *rlog* and VST in the ‘DESeq2’ package did not reproduce the correct grouping of biological replicates. (TIFF 694 kb)

Additional file 8: In silico reference genes predicted on including two conditions. To examine how the choice of in silico invariant genes depends on the choice of conditions, we applied *moose* ^*2*^ to subsets consisting of two conditions each. While the number of predictions increases when including only two conditions (0/30, 0/90, and 30/90), there are a number of predicted genes shared among the data sets. Grey shading in the table indicates the six established reference genes. The Venn diagram visualizes overlaps between predictions. (PDF 78 kb)

Additional file 9: Contribution of in silico predictions and quadratic correction. Shown are the sample groupings for default parameters, as used in our experiment (a); grouping using a linear fit (b); and no predictions and quadratic fit (c). Out of these, only the combination of predictions and quadratic fit achieve the correct grouping. Also shown are the results when supplying a list of six differently expressed (DE) genes as reference genes (d), in which case five genes are rejected; six randomly selected genes also resolve the grouping correctly (e). (TIFF 1271 kb)

Additional file 10: DESeq2 analyses in different modes. Supplying DESeq2 with the *moose* ^*2*^ predictions does not accurately resolve the sample grouping. (TIFF 392 kb)

Additional file 11: Relative log expression (RLE) boxplots for *moose* ^*2*^-normalized data. Centering on zero and/or variance are improved when in silico predictions are based on a set of established reference genes. (TIFF 494 kb)

Additional file 12: Correlation plots for invariant genes. Genes, that were predicted by *moose* ^*2*^ to be expression-invariant, were correlated with each other according to their transcript counts across all RNA-seq samples. (A) Raw read counts (no normalization) and (B) DESeq2-normalized read counts. The scale bar depicts Pearson’s Rho. (TIFF 6253 kb)

Additional file 13: Correlation plots for invariant genes. Genes, that were predicted by *moose* ^*2*^ to be expression-invariant, were correlated with each other according to their transcript counts across all RNA-seq samples. (A) Raw read counts (no normalization) and (B) *moose* ^*2*^-normalized read counts. The scale bar depicts Pearson’s Rho. (TIFF 6341 kb)

Additional file 14: Correlations of gene expression changes for 19 selected genes. Log_2_ ratios derived from five normalization methods (RPKM, UQ, TMM, DESeq2, and *moose* ^*2*^) are compared to qRT-PCR measurements for 30-to-0-min (upper panel) and 90-to-0-min comparisons (lower panel). *R*, Pearson’s rho. (TIFF 655 kb)

Additional file 15: Coverage curve for LexA-dependent genes. *E. coli* MG1655 genes were sorted according to their *p*-values, starting with the lowest value. The Top-1000 list (grey box, *p*-value cutoff: *p* < 9.563*10^−4^) includes 31 out of 58 LexA-dependent genes. (TIFF 426 kb)

Additional file 16: MA-plots for *moose* ^*2*^-normalized RNA-seq data. The average of expression values (log_2_-transformed read counts) is plotted on the x-axis, and the y-axis shows log_2_ ratios between conditions. Every dot represents one gene. Differentially expressed genes (DEGs) were determined using *Limma* (*p* < 9.563*10^−4^) and are shown in red. (TIFF 688 kb)
