# Supplementary material for: RNA-sequence data normalization through in silico prediction of reference genes: the bacterial response to DNA damage as case study
Source: BioData Min. 2017 Sep 5;10:30. doi: 10.1186/s13040-017-0150-8 (PMC5584328; doi:10.1186/s13040-017-0150-8)
Supplement: Supplementary file 1 — Primers used for qRT-PCR. (PDF 18 kb) [file 13040_2017_150_MOESM1_ESM.pdf]

Additional file 1. Primers used for qRT-PCR.

Name	Sequence (5' to 3')
**Reference genes**	
cysG-1	taaccagcgcgtcagcgaag
cysG-2	catgagcggtgagcggtcaa
idnT-1	ggcgtcgcgctgcttcttat
idnT-2	aggacggcctgtgcatccat
hcaT-1	atccgtccgacgattcag
hcaT-2	ccgtaataggccgcatgt
ihfB-1	gcatatggcctcgactcttg
ihfB-2	attacgtccggtacgtggtg
ssrA-1	ggcaagcgaatgtaaagactga
ssrA-2	ccgcgtccgaaattccta
rrsA-1	agagcaagcggacctcataa
rrsA-2	gtattcaccgtggcattctg
**LexA-dependent genes**	
ftsK-1	tgaactggcgtcttacgg
ftsK-2	agcgttgctgctgtgtct
dinG-1	ggtctgcgcgatcatact
dinG-2	tccgcttcctgaatctcc
cho-1	gcagaccatcgctgatga
cho-2	agcgtagcgcatgttcct
yebG-1	atacggcggatctgcttg
yebG-2	attcagcaccgacgacct
uvrD-1	cgcatatcctgcaacact
uvrD-2	tcaccgacgatcatcact
uvrB-1	cgaggaacacggaattac
uvrB-2	cacattatccggctcaac
recN-1	catccggtggtgaattgt
recN-2	agttgattcgccgagttg
polB-1	cgcaacgagccatatcag
polB-2	catgaggcggcacattac
**Randomly selected genes**	
ahpC-1	ctgaaggcattggccgtgac
ahpC-2	ccagagacggagccagagtt
can-1	ggattggatgttccgacagt
can-2	gtgtgaatgaccaggttagc
cspE-1	cacttctctgcaatccagac
cspE-2	gctgaccttcagcaagagtt
cyoA-1	ggcattgctaccgtgaatga
cyoA-2	agacgcggaatgaagaagga
gatY-1	tctggcgtgcgctcagtcat
gatY-2	cgcctcgacgctgacatcaa
gatZ-1	tgaatcttgaaggcgtggac
gatZ-2	agctgatgcggtattgctga
map-1	cgagctggatcgcatctgta
map-2	ttcggatagccgtgatagcc
sodA-1	tggcggtcacgctaaccaca
sodA-2	tcaacggagccgaagtcacg
ubiD-1	ccacgattctcggtgcagtc
ubiD-2	tcggtcttggtgccacgtag
yajC-1	ccacagcagaagcgcaccaa
yajC-2	aaccaggccaccgttcgtca
yggX-1	acatgatgaatgccgagcac
yggX-2	ttgaccatctcctgctcaag
