# Supplementary material for: RNA-sequence data normalization through in silico prediction of reference genes: the bacterial response to DNA damage as case study
Source: BioData Min. 2017 Sep 5;10:30. doi: 10.1186/s13040-017-0150-8 (PMC5584328; doi:10.1186/s13040-017-0150-8)
Supplement: Supplementary file 2 — Number of in silico invariant genes depending on the choice of parameters. The number of genes depends on h and m, with several settings resulting in the same set of 33 genes used in this analysis. (DOCX 8 kb) [file 13040_2017_150_MOESM2_ESM.docx]

|  | h=1.0 | h=2.0 | h=3.0 | h=4.0 | h=5.0 | h=6.0 | h=7.0 |
| --- | --- | --- | --- | --- | --- | --- | --- |
| m=1.0 | 32 | 29 | 29 | 29 | 29 | 29 | 29 |
| m=2.0 | 51 | 34 | **33** | 31 | 31 | 31 | 31 |
| m=3.0 | 123 | 46 | 34 | **33** | **33** | 31 | 31 |
| m=4.0 | 620 | 53 | 43 | 34 | **33** | **33** | **33** |
| m=5.0 | 1541 | 84 | 50 | 43 | 34 | **33** | **33** |
| m=6.0 | 2310 | 161 | 53 | 46 | 43 | 34 | **33** |

**Number of predicted *in silico* invariant genes depending on the HMM parameters *h* (columns) and *m* (rows)**. Based on 33 being the most common result with these values tested, all sets of 33 being identical, and all other sets being sub- or super-sets, we chose m=4.0 and h=5.0.
