# Supplementary figures and images for: RNA-sequence data normalization through in silico prediction of reference genes: the bacterial response to DNA damage as case study
Source: BioData Min. 2017 Sep 5;10:30. doi: 10.1186/s13040-017-0150-8 (PMC5584328; doi:10.1186/s13040-017-0150-8)

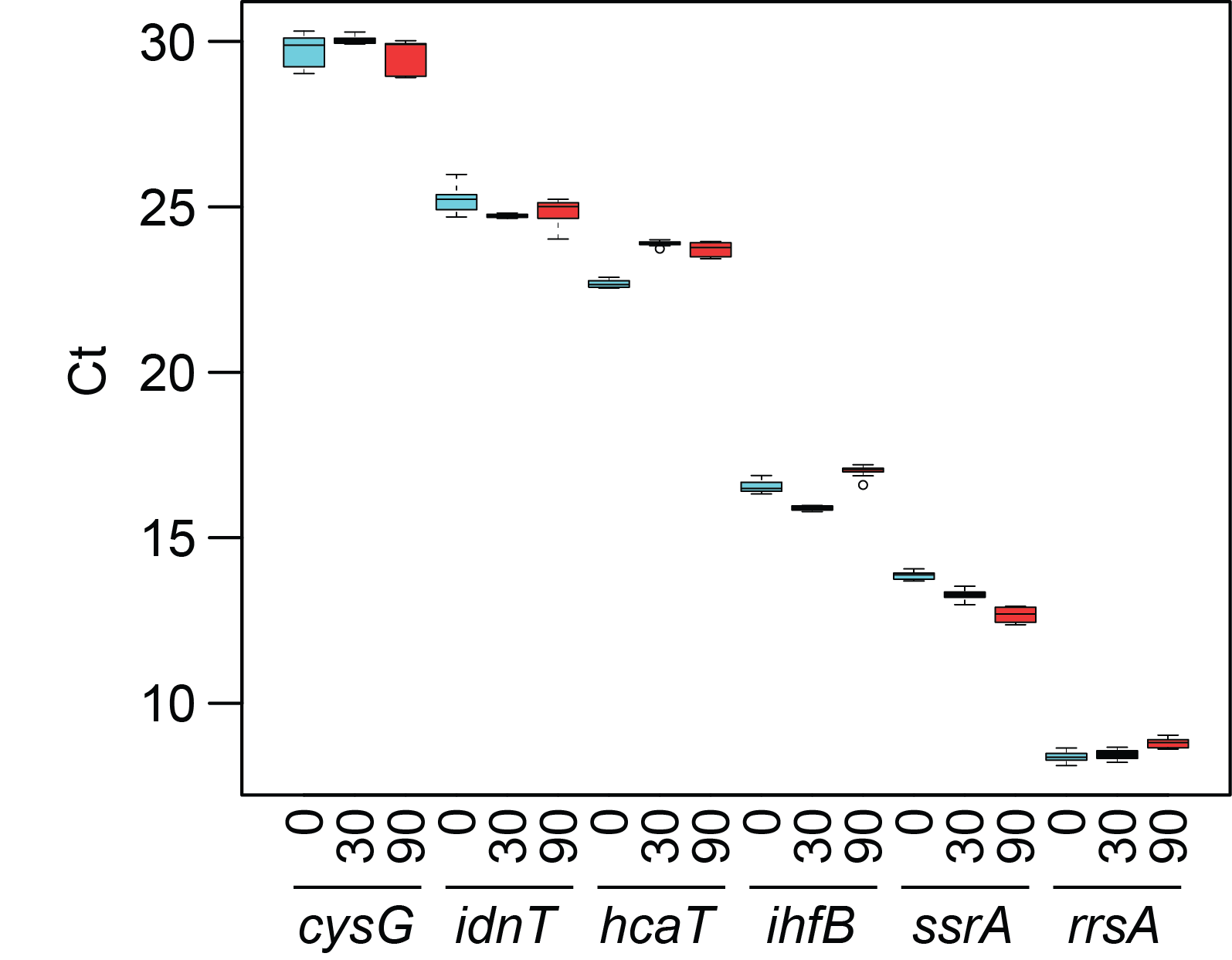

Supplement: Supplementary file 6 — Box plots of reference genes broken down by condition. While there are minor differences in expression levels, there is no systematic trend in either direction. (TIFF 238 kb) [file 13040_2017_150_MOESM6_ESM.tif]

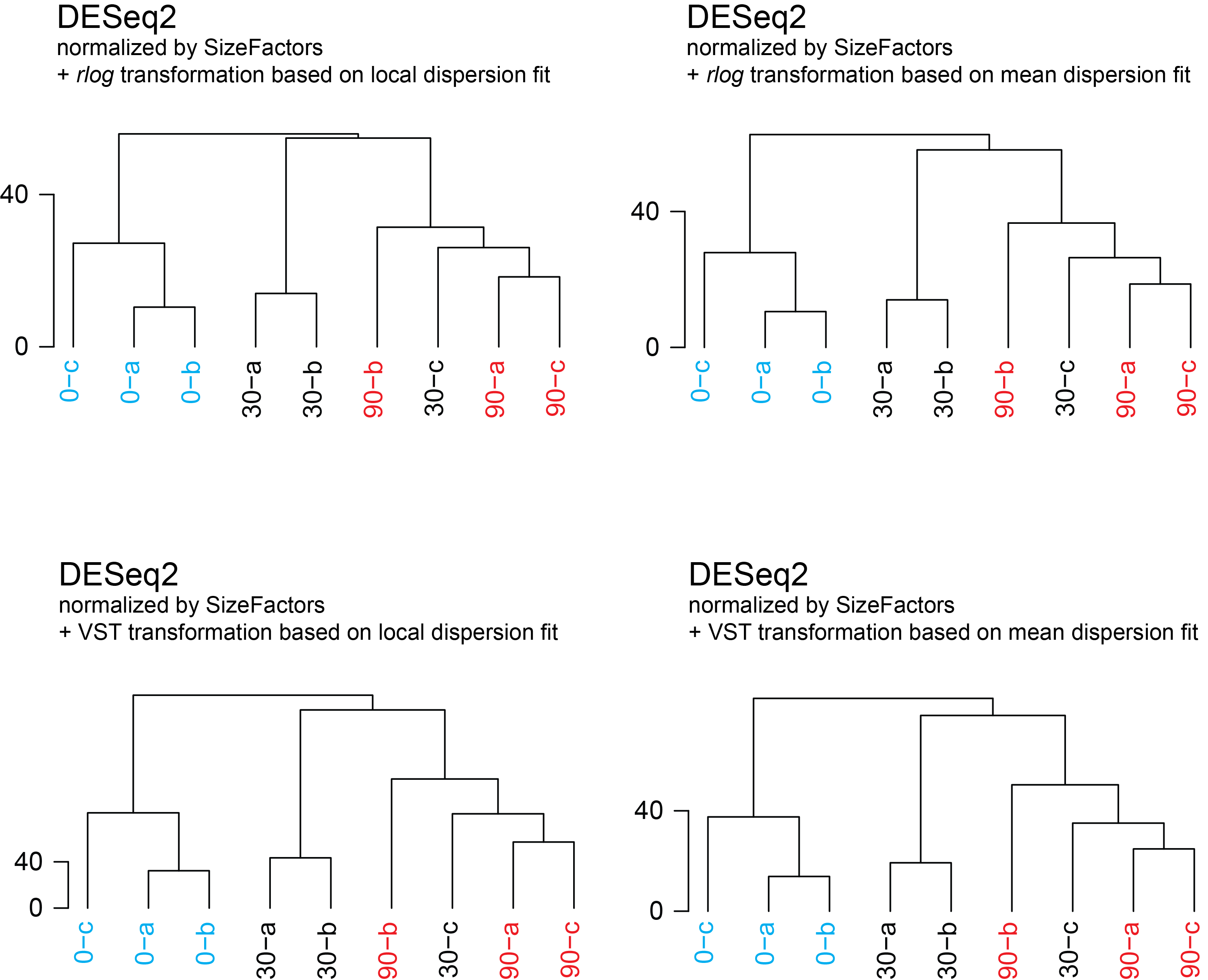

Supplement: Supplementary file 7 — Per-gene dispersion estimates for the log-transformation. Using rlog and VST in the ‘DESeq2’ package did not reproduce the correct grouping of biological replicates. (TIFF 694 kb) [file 13040_2017_150_MOESM7_ESM.tif]

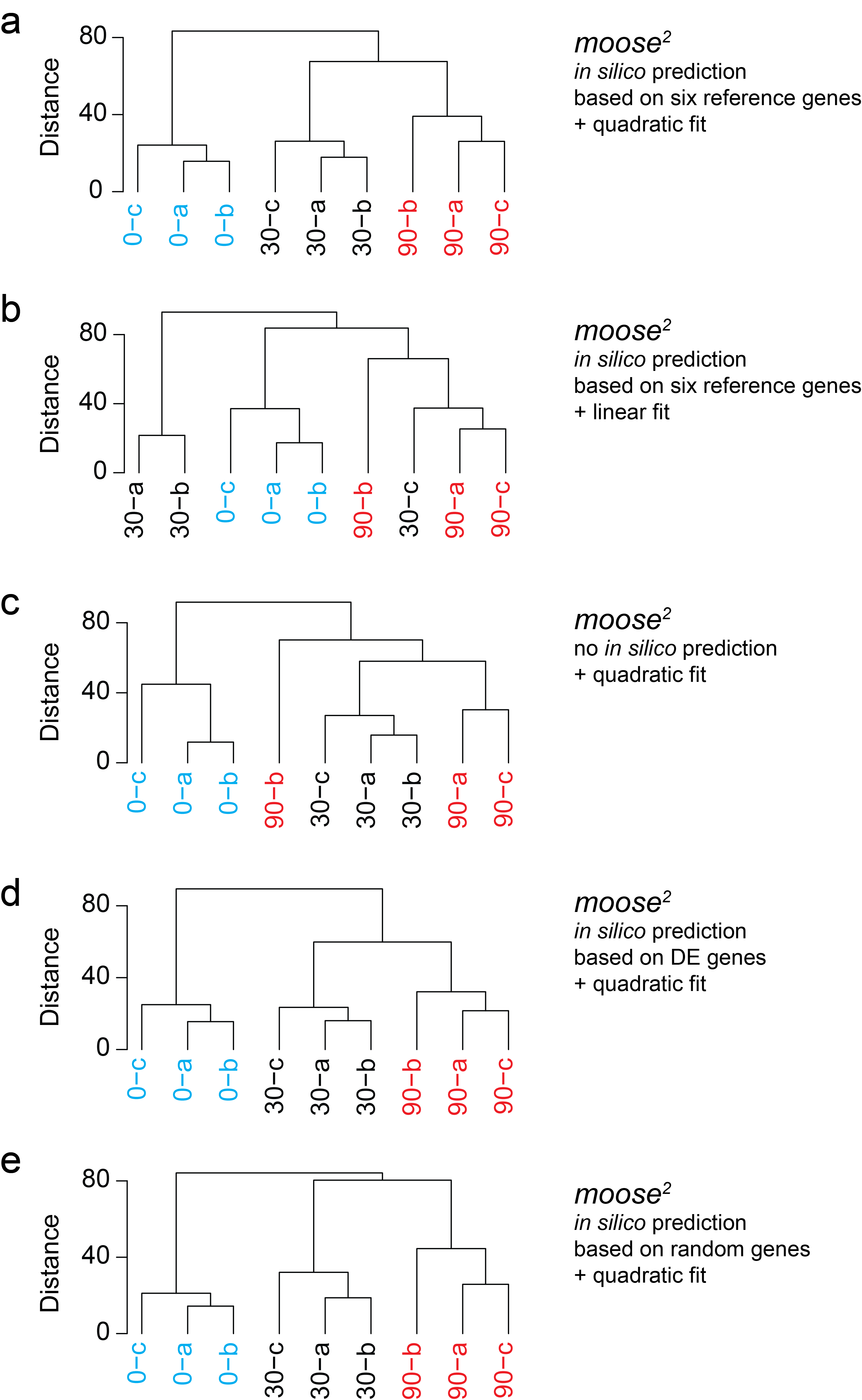

Supplement: Supplementary file 9 — Contribution of in silico predictions and quadratic correction. Shown are the sample groupings for default parameters, as used in our experiment (a); grouping using a linear fit (b); and no predictions and quadratic fit (c). Out of these, only the combination of predictions and quadratic fit achieve the correct grouping. Also shown are the results when supplying a list of six differently expressed (DE) genes as reference genes (d), in which case five genes are rejected; six randomly selected genes also resolve the grouping correctly (e). (TIFF 1271 kb) [file 13040_2017_150_MOESM9_ESM.tif]

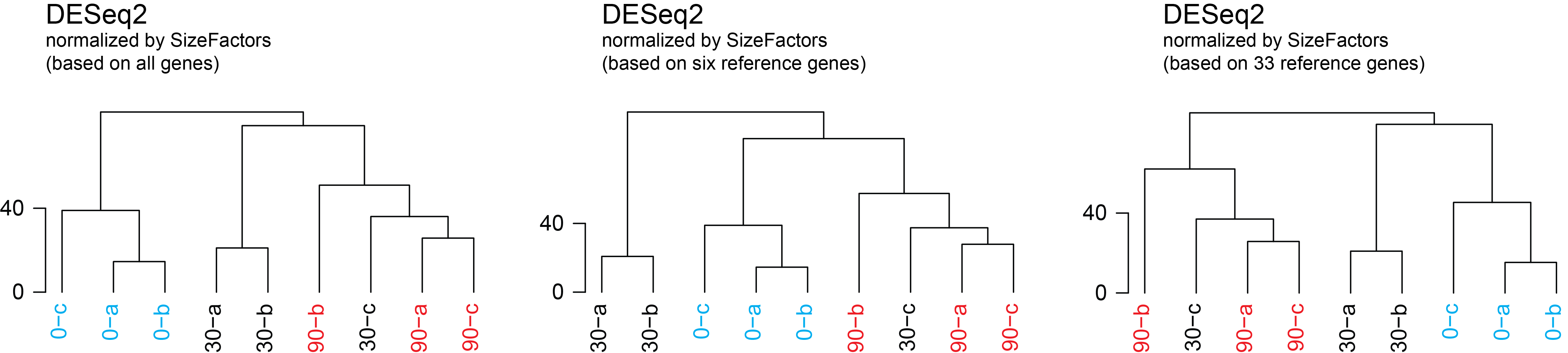

Supplement: Supplementary file 10 — DESeq2 analyses in different modes. Supplying DESeq2 with the moose 2 predictions does not accurately resolve the sample grouping. (TIFF 392 kb) [file 13040_2017_150_MOESM10_ESM.tif]

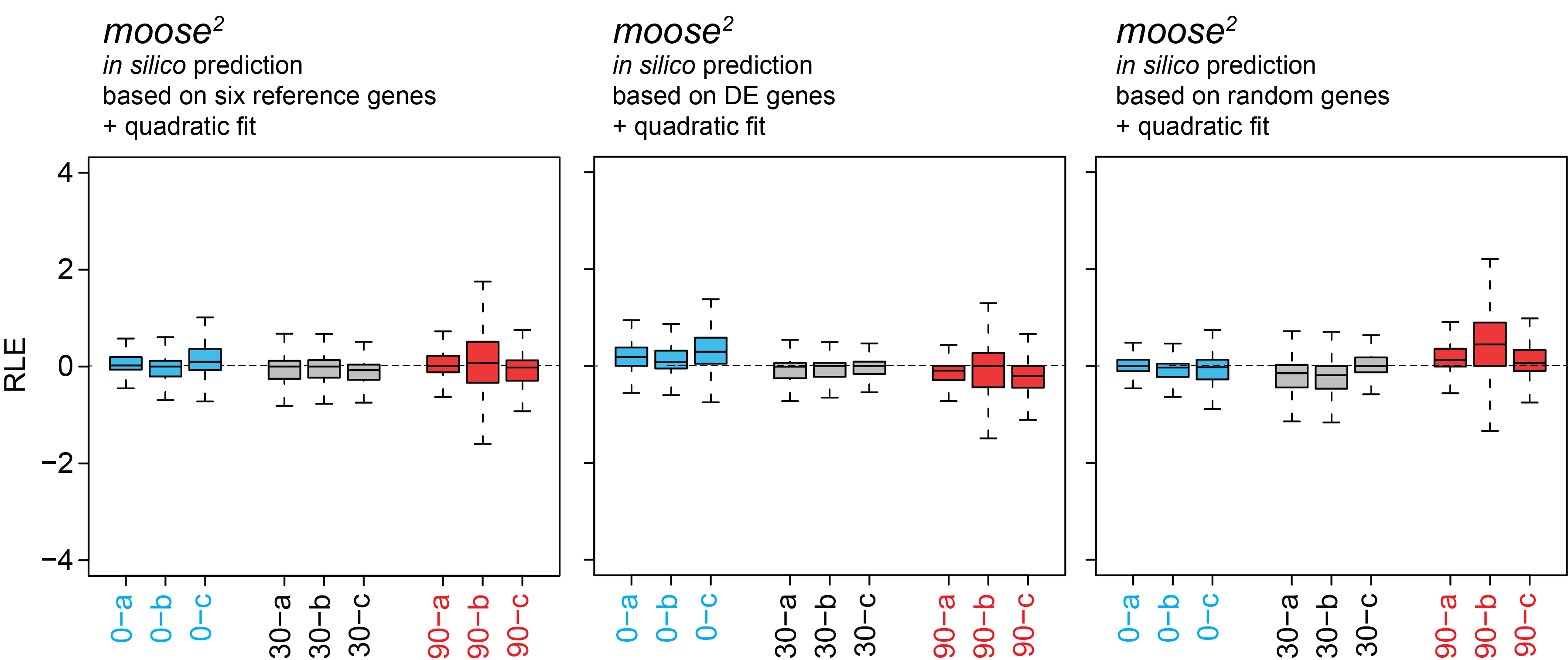

Supplement: Supplementary file 11 — Relative log expression (RLE) boxplots for moose 2-normalized data. Centering on zero and/or variance are improved when in silico predictions are based on a set of established reference genes. (TIFF 494 kb) [file 13040_2017_150_MOESM11_ESM.tif]

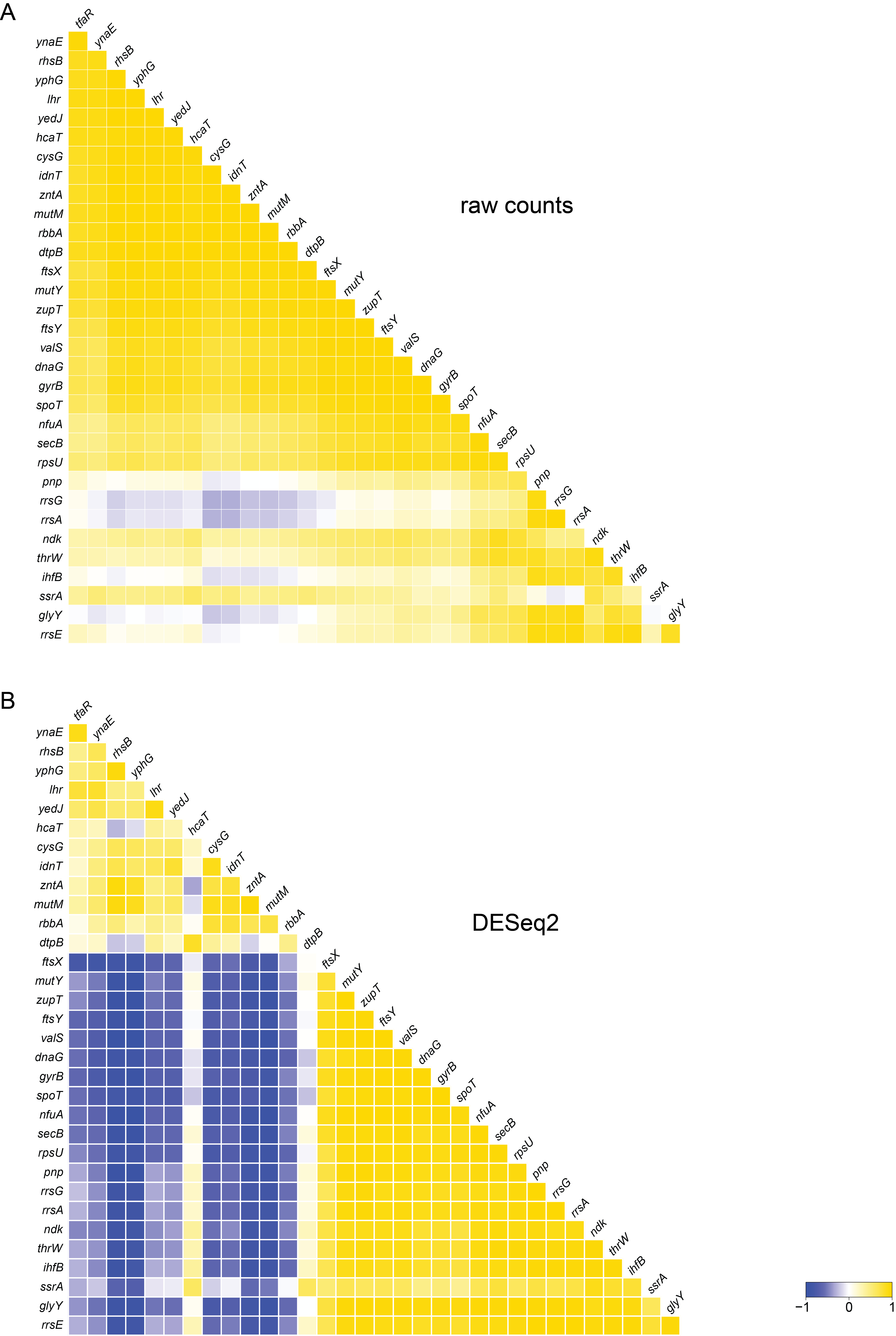

Supplement: Supplementary file 12 — Correlation plots for invariant genes. Genes, that were predicted by moose 2 to be expression-invariant, were correlated with each other according to their transcript counts across all RNA-seq samples. (A) Raw read counts (no normalization) and (B) DESeq2-normalized read counts. The scale bar depicts Pearson’s Rho. (TIFF 6253 kb) [file 13040_2017_150_MOESM12_ESM.tif]

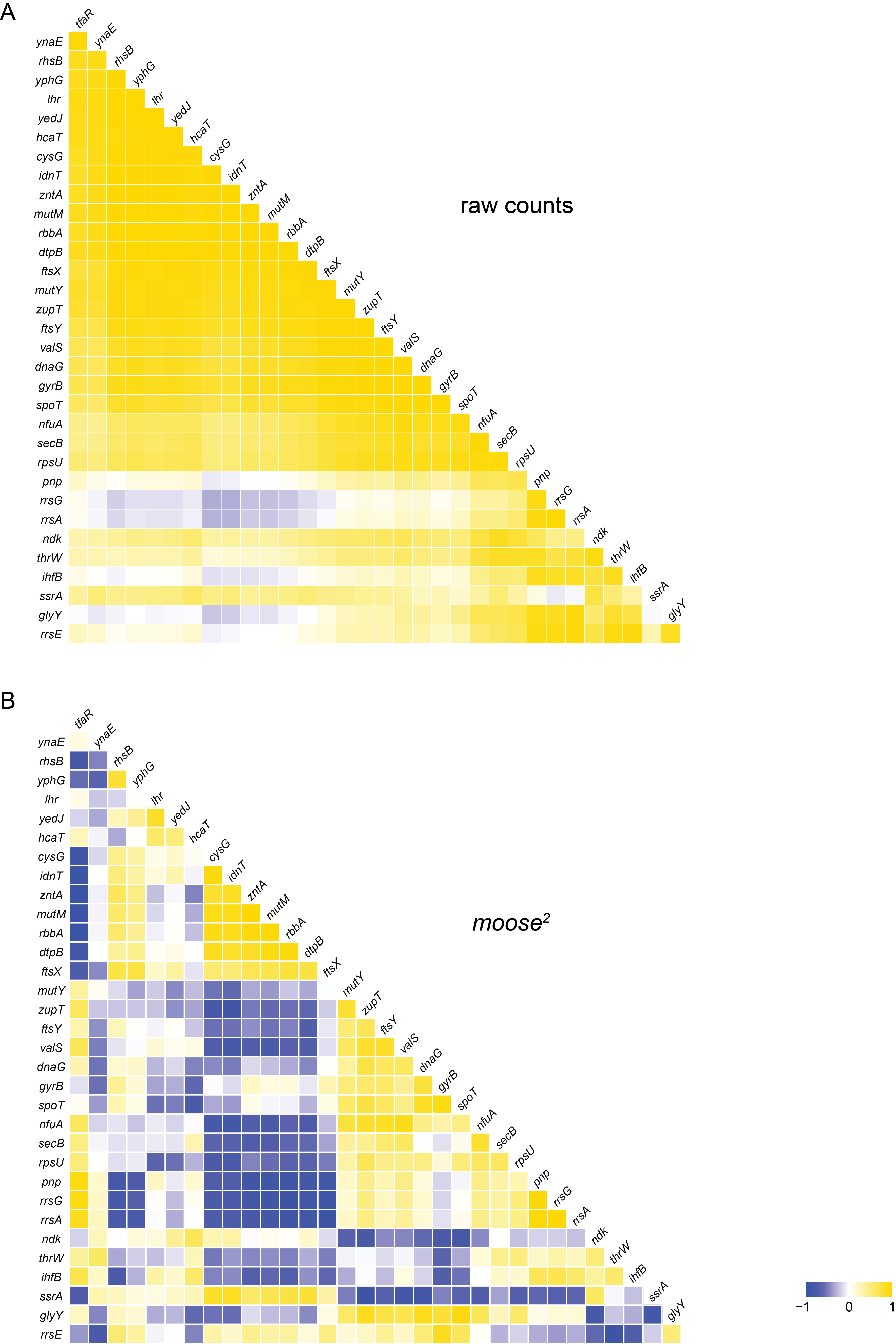

Supplement: Supplementary file 13 — Correlation plots for invariant genes. Genes, that were predicted by moose 2 to be expression-invariant, were correlated with each other according to their transcript counts across all RNA-seq samples. (A) Raw read counts (no normalization) and (B) moose 2-normalized read counts. The scale bar depicts Pearson’s Rho. (TIFF 6341 kb) [file 13040_2017_150_MOESM13_ESM.tif]

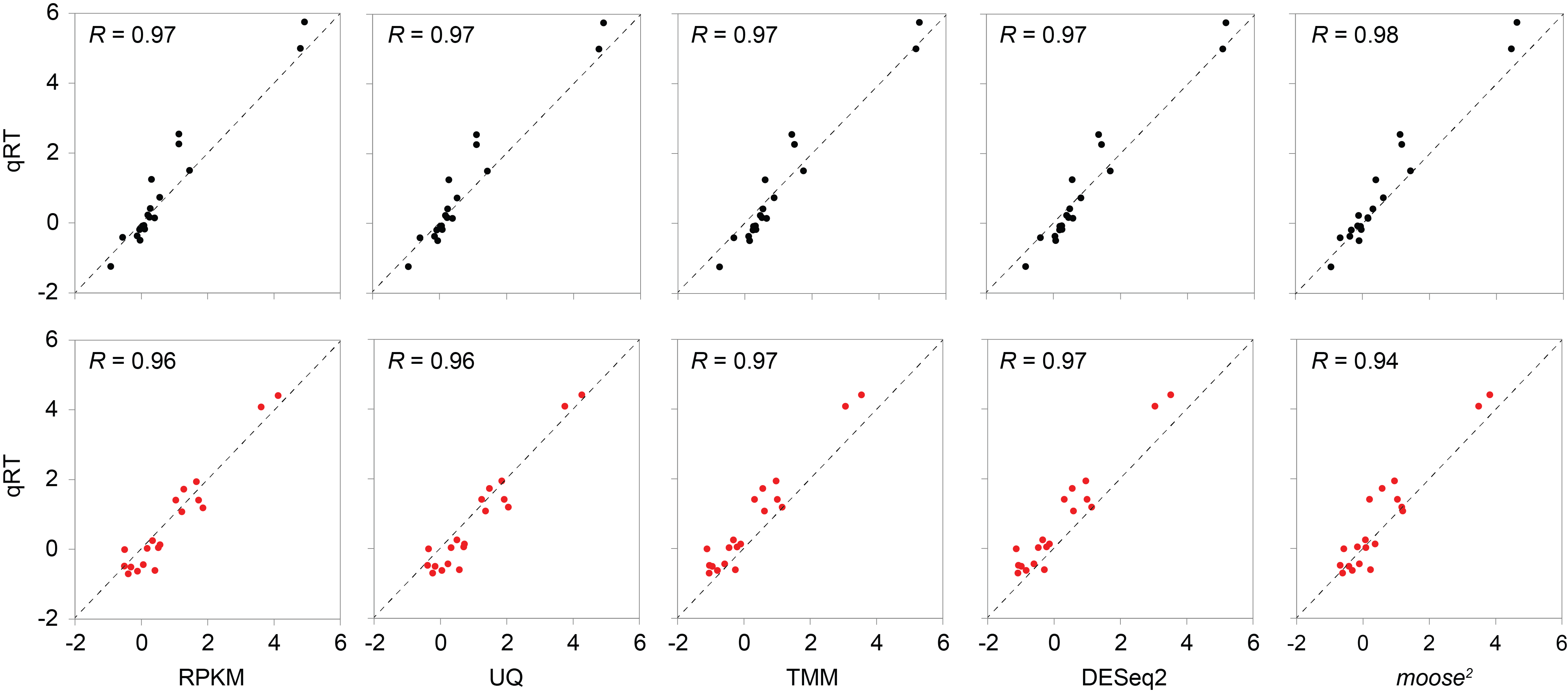

Supplement: Supplementary file 14 — Correlations of gene expression changes for 19 selected genes. Log2 ratios derived from five normalization methods (RPKM, UQ, TMM, DESeq2, and moose 2) are compared to qRT-PCR measurements for 30-to-0-min (upper panel) and 90-to-0-min comparisons (lower panel). R, Pearson’s rho. (TIFF 655 kb) [file 13040_2017_150_MOESM14_ESM.tif]

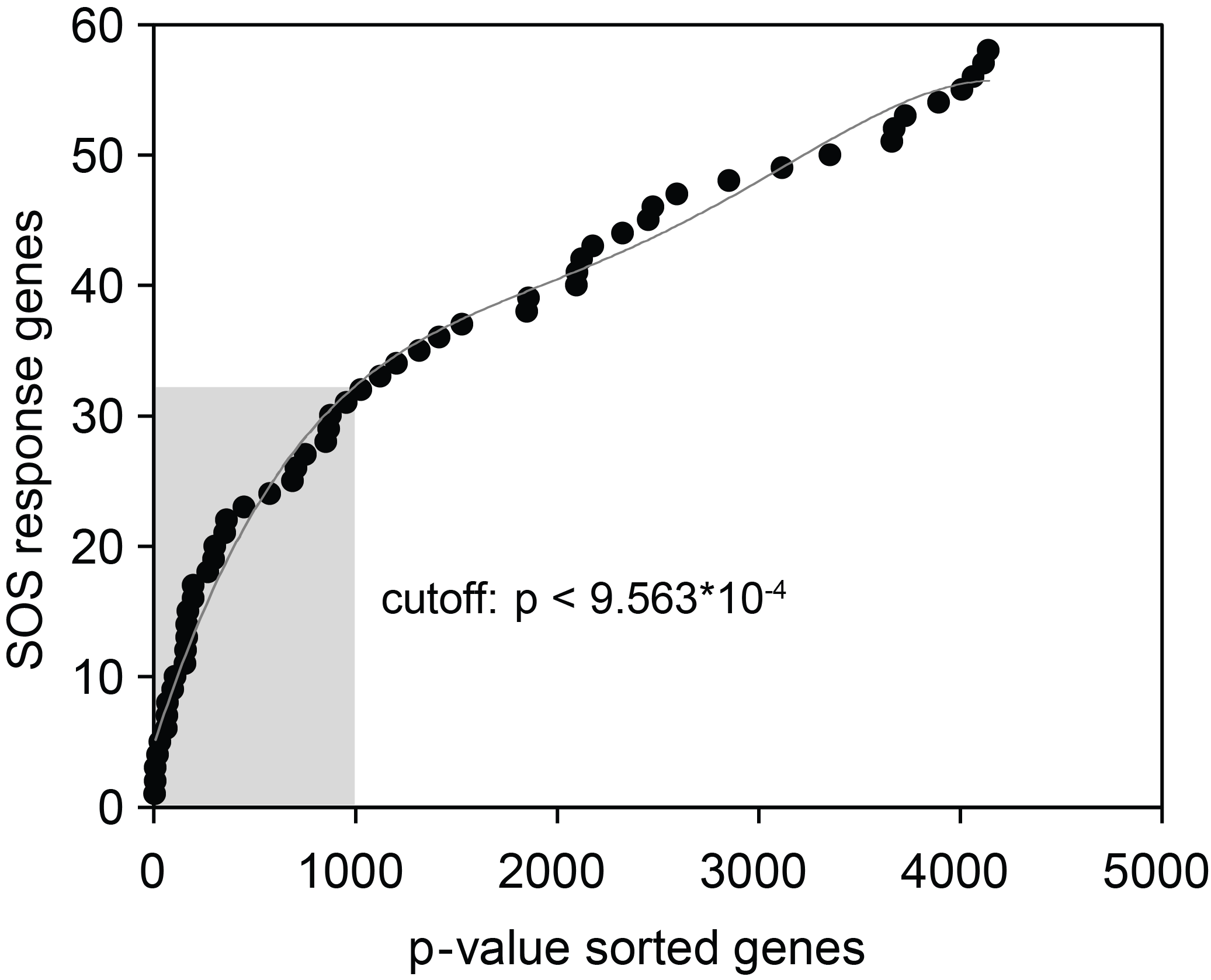

Supplement: Supplementary file 15 — Coverage curve for LexA-dependent genes. E. coli MG1655 genes were sorted according to their p-values, starting with the lowest value. The Top-1000 list (grey box, p-value cutoff: p < 9.563*10−4) includes 31 out of 58 LexA-dependent genes. (TIFF 426 kb) [file 13040_2017_150_MOESM15_ESM.tif]

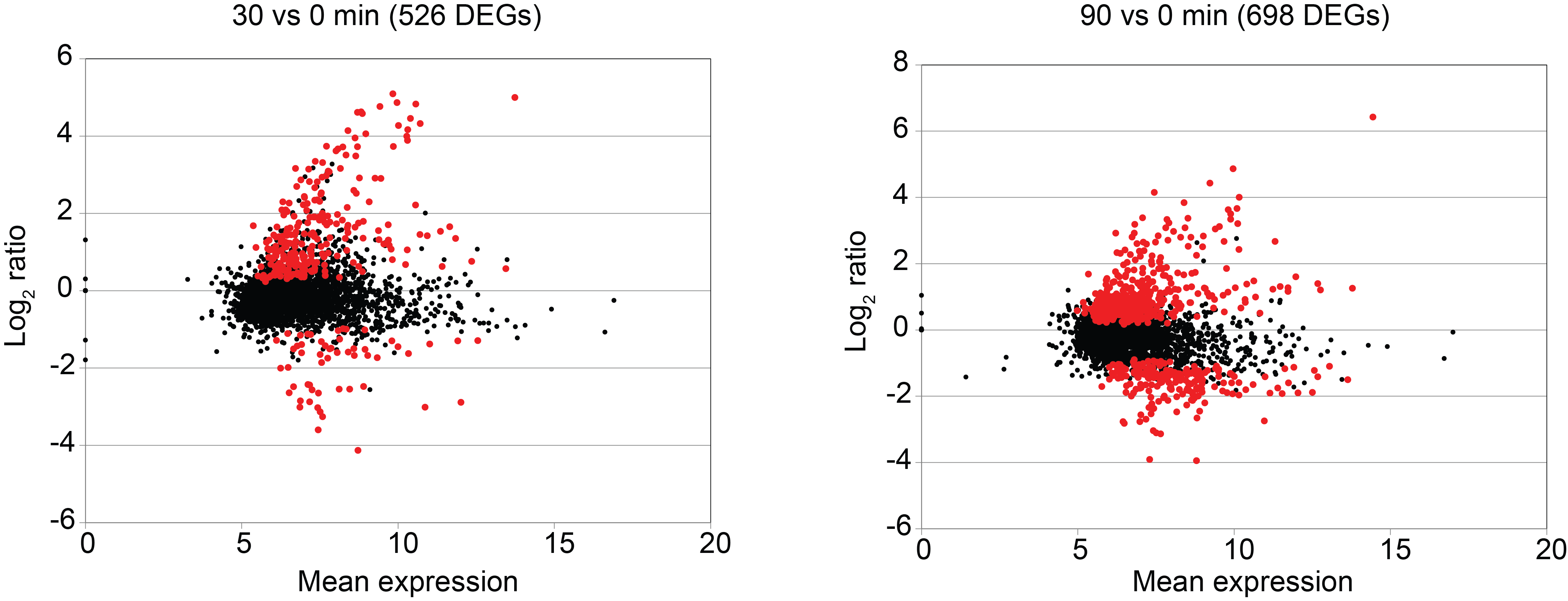

Supplement: Supplementary file 16 — MA-plots for moose 2-normalized RNA-seq data. The average of expression values (log2-transformed read counts) is plotted on the x-axis, and the y-axis shows log2 ratios between conditions. Every dot represents one gene. Differentially expressed genes (DEGs) were determined using Limma (p < 9.563*10−4) and are shown in red. (TIFF 688 kb) [file 13040_2017_150_MOESM16_ESM.tif]
