# Supplementary material for: RNA-sequence data normalization through in silico prediction of reference genes: the bacterial response to DNA damage as case study
Source: BioData Min. 2017 Sep 5;10:30. doi: 10.1186/s13040-017-0150-8 (PMC5584328; doi:10.1186/s13040-017-0150-8)

0/30/90	0/30	0/90	30/90
**cysG**	acrA	alsR	betT
dnaG	asnC	aroK	ccmA
dtpB	cof	atpA	chbC
ftsX	**cysG**	crfC	crp
ftsY	envZ	cyoC	**cysG**
glyY	fadL	**cysG**	dtpB
gyrB	fbp	eno	efp
**hcaT**	fmt	exuR	fabF
**idnT**	gatA	fecC	fbp
**ihfB**	gatY	fimA	ftsX
lhr	glyY	fimH	gatZ
mutM	**hcaT**	focA	gcvA
mutY	hldD	ftsY	gshB
ndk	hyfA	gatZ	**hcaT**
nfuA	**idnT**	gnd	lcd
pnp	**ihfB**	gss	**idnT**
rbbA	ileT	**hcaT**	**ihfB**
rhsB	insG	Hcr	infB
rpsU	kdpB	hokB	melA
**rrsA**	lpxC	**idnT**	ompR
rrsE	mlaC	**ihfB**	paaF
rrsG	mltD	intA	pgaC
secB	nagC	iscX	pgk
spoT	ndk	leuW	pnp
**ssrA**	nfuA	mutM	prfB
tfaR	nrdD	mutY	priB
thrW	nrdR	nanC	rho
valS	nudF	ogrK	rhsB
yedJ	pepD	oppB	rlmJ
ynaE	pgk	pabC	rpiA
yphG	plsB	pfkA	rplJ
zntA	rhaM	pldA	rplN
zupT	rho	prmB	rpoA
	rhsB	qorB	rrlB
	rpiA	rhsB	**rrsA**
	rpsF	**rrsA**	speF
	rpsG	rrsG	**ssrA**
	rpsO	rzpQ	surA
	**rrsA**	sdsR	tfaR
	rrsC	sodA	thiF
	rrsE	speA	tisB
	rutF	**ssrA**	tyrS
	sdaA	ssuD	uspA
	**ssrA**	tolC	uvrA
	tfaR	ycfZ	waaQ
	trmA	ydeN	yaeF
	valS	ydiQ	ybhH
	ydhK	ydjY	ybjL
	yeaW	yejK	yeaC
	yfbT	ygiB	yeaX
	yfcC	yheS	yfeX
	ygfB	yhhJ	yhiI
	yhfK	zupT	yjjK
	yidB		ynaE
	yifB		ypdC
	yigA		ypdE
	ynaE		yrbL
			yrdD
33	57	53	58

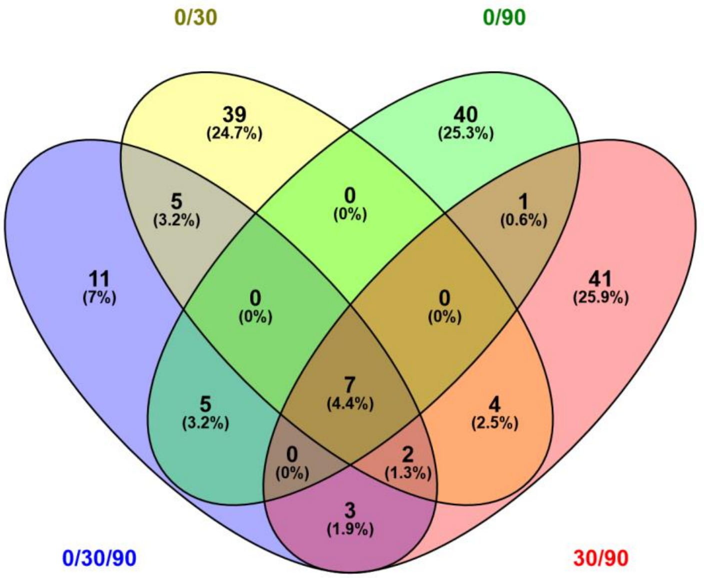

Supplement: Supplementary file 8 — In silico reference genes predicted on including two conditions. To examine how the choice of in silico invariant genes depends on the choice of conditions, we applied moose 2 to subsets consisting of two conditions each. While the number of predictions increases when including only two conditions (0/30, 0/90, and 30/90), there are a number of predicted genes shared among the data sets. Grey shading in the table indicates the six established reference genes. The Venn diagram visualizes overlaps between predictions. (PDF 78 kb) [file 13040_2017_150_MOESM8_ESM.pdf]
